# Tumor-suppressive microRNA-152 inhibits the proliferation of Ewing’s sarcoma cells by targeting CDK5R1

**DOI:** 10.1038/s41598-023-45833-6

**Published:** 2023-10-29

**Authors:** Masanori Kawano, Kazuhiro Tanaka, Ichiro Itonaga, Tatsuya Iwasaki, Yuta Kubota, Hiroshi Tsumura

**Affiliations:** Department of Orthopaedic Surgery, Faculty of Medicine, Oita University, Oita, 879-5593 Japan

**Keywords:** Oncology, Cancer, Bone cancer

## Abstract

We elucidated the mechanism through which the reduced expression of miR-152 leads to the overexpression of its target cyclin-dependent kinase-5 activator 1 (CDK5R1) in Ewing’s sarcoma (ES) cells and the role of this mechanism in the proliferation of ES cells. To explore possible oncogenic factors in ES, we conducted microarray-based investigation and profiled the changes in miRNA expression and their effects on downstream mRNAs in five ES cell lines and human mesenchymal stem cells (hMSCs). miR-152 was significantly downregulated, while cyclin-dependent kinase-5 activator 1 (CDK5R1) expression was significantly upregulated in all tested ES cells as compared to hMSCs. The overexpression of CDK5R1 led to the activation of CDK5, enabling the phosphorylation of retinoblastoma protein and persistent overexpression of CCNE. Moreover, miR-152 suppressed cell proliferation via cell cycle retardation, and its upregulation reduced tumor size and CCNE expression in tumor tissues. The overexpression of cyclin E (CCNE) has been detected in ES cells, but the detailed mechanisms have not been previously elucidated. These findings identify the miR152-CDK5R1 signaling axis as a critical mechanism for tumorigenesis that may serve as a new therapeutic target in Ewing’s sarcoma. We believe that our results will aid in the development of effective treatment strategies for patients with ES.

## Introduction

Ewing’s sarcoma (ES) is the second most common primary malignant bone tumor in children and young adults that has a peak incidence at age 15^1^. Despite advances in therapy, up to 40% of patients with ES eventually relapse^2^. ES tumors are difficult to control and thus there is a need for new treatment options that are equivalent to or better than the standard treatment^3^.

MicroRNAs (miRNAs) are a large family of noncoding single-stranded RNAs of 18–25 nucleotides that play an important role in post-transcriptional regulation^4, 5^. Aberrant expression of miRNAs has been widely studied in human cancers, including bone cancer^6^. We explored genome wide array expression of both miRNAs and mRNAs in human mesenchymal stem cells (hMSCs) and five human ES cell lines. The results showed that the expression of miR-152 was decreased whereas that of CDK5R1 was upregulated in five ES cell lines compared to hMSCs. A tumor-suppressive miRNA, miR-152, has been reported in other cancers^7, 8^. CDK5R1, also known as p35, has been identified as a tumor promoter^9^. However, the relationship between miR-152 and its target cyclin dependent kinase 5 activator 1 (CDK5R1) in ES has not been previously elucidated.

CDK5R1 is activated when calpain protein cleaves p35 into p25, and this forms a complex with cyclin-dependent kinase 5 (CDK5) in the cytoplasm, which is then translocated into the nucleus to promote cell proliferation^10^. CDK5 plays an important role in central nervous system development and function maintenance^11, 12^. Abnormal expression of CDK5 has been detected in certain cancers, and it may be significantly involved in the progression of malignant tumors^9, 13^. Furthermore, CDK5 enhances the expression of cyclin E (CCNE) by promoting transcription via E2F protein through phosphorylation of retinoblastoma (Rb) proteins^14, 15^. Thus, CDK5R1 overexpression may be related to CCNE overexpression.

The aim of this study was to elucidate the mechanism by which overexpression of CDK5R1 due to reduced expression of miR-152 promotes the progression of the cell cycle through phosphorylation of Rb proteins and chronic upregulation of CCNE. Our result will elucidate the mechanism by which high expression of CDK5R1 due to reduction of miR-152 leads to cell-cycle progression through the phosphorylation of Rb protein and the constitutive upregulation of CCNE.

## Results

### Significantly reduced expression of miR-152 and overexpression of CDK5R1 were observed in all ES cell lines

Comprehensive microarray analysis showed reduced expression of miR-152in five different ES cell lines compared with hMSCs (2.63–40.21 times) (Supplemental Fig. S1a). The cDNA array showed overexpression of CDK5R1 in five ES cell lines compared with hMSCs (2.69–6.55 times) (Supplemental Fig. S1b).

### miR-152 directly targeted the 3′-UTR of CDK5R1

**Figure 1:**
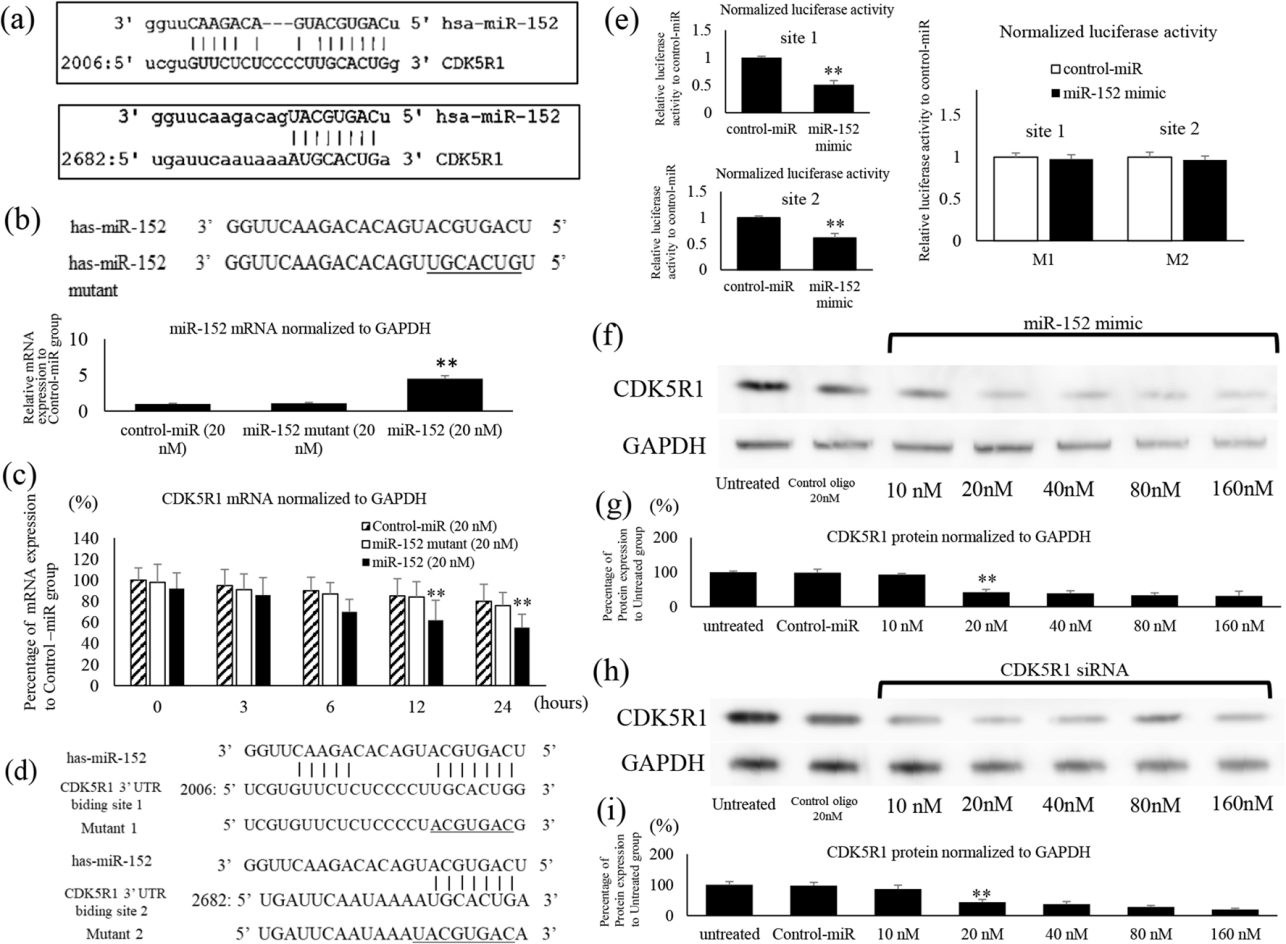
CDK5R1 is the target gene of miR-152. (**a**) The binding site of CDK5R1 and miR-152 is shown. (**b**) The sequence of miR-152 and the sequence of miR-152 mutant with mutation in the seeded region. After transfection of SKES-1 cells, miR-152 was detected by quantitative PCR from RNA extracted from the cells. miR-152 was appropriately introduced into the cells, and the miR-152 mutant did not differ from the control group. (**c**) Intracellular expression of CDK5R1 after transfection with miR-152 or miR-152 mutant as analyzed by reverse transcriptase-quantitative polymerase chain reaction (qRT-PCR). (**d**) Predicted binding sites of miR-152 in the 3′-untranslated region (UTR) of CDK5R1 binding sites 1 and 2. Sites mutated as controls for the luciferase assay are underlined. WT, wild-type; M1, mutation of CDK5R1 3′-UTR binding site 1; M2, mutation of CDK5R1 3′-UTR binding site 2. (**e**) Luciferase assay identified CDK5R1 as two targets of miR-152. Luciferase reporter assay for the direct and specific interaction of miR-152 with predicted two target sites in the 3′-UTR of CDK5R1. Reporter activity was significantly reduced in miR-152 mimic transfected cells. Control-miR cells were used as control here for normalization. The mutations at the two target sites did not reduce luciferase activity in response to the miR-152 mimic; it remained equivalent to the Control-miR group. Images (**f**) and plots (**g**) from the immunoblot analysis of CDK5R1 protein expression following changes in miR-152 expression. Images (**h**) and plots (**i**) of protein expression of CDK5R1 following changes in the concentration of CDK5R1 siRNA. GAPDH level was used as loading control.

Two possible binding sites of miR-152 and 3′-UTR of CDK5R1 were predicted using BLAST (Fig. 1a) and Target Scan 8.0 (https://www.targetscan.org/vert_80/) (Supplemental Fig. S2). Oligonucleotides with sequence mutations in the seed region of miR-152 were created and transfected into ES cell lines (Fig. 1b). In comparison with the control-miR and miR-152 mutant groups, overexpression of miR-152 was observed only in the miR-152-transfected group (p < 0.01). Overexpression of miR-152 was confirmed in cells transfected with oligonucleotides (miR-152 mutant), thereby showing that the transfection was successful. To determine whether miR-152 targeted CDK5R1, we evaluated the levels of CDK5R1 mRNA. Compared with cells transfected with control-miR and miR-152 mutant, the cells transfected with oligonucleotide miR-152 mimic showed significantly reduced expression of CDK5R1 (p < 0.01; Fig. 1c). To clarify the direct target of miR-152, sites 1 and 2 of the 3′-UTR of CDK5R1 mRNA transcript were inserted separately into a dual-luciferase vector pmirGLO, and the ability of miR-152 to regulate the reporter gene was examined (Fig. 1d). Addition of miR-152 mimic resulted in significant repression of sites 1 and 2 compared to that in control-miR (100%). Further, we confirmed the direct and specific regulation of CDK5R1 3′-UTR binding site mutations (sites 1 and 2) by miR-152 mimic using a luciferase reporter assay. There were no significant differences between mutant site 1 and 2 and control-miR (Fig. 1e).

Thus, our results showed that the 3′-UTR of CDK5R1 was a target of miR-152. It was found that successfully transfected miR-152 targets mRNA. Therefore, we then examined whether miR-152 reduced the expression of the CDK5R1 protein (Fig. 1f).

The quantification of protein expression showed a reduced expression of CDK5R1 in cells transfected with 20 nM miR-152 mimic compared with the untreated cells (Fig. 1g). Protein expression after transfection with CDK5R1 siRNA was observed (Fig. 1h). The transfection with CDK5R1 siRNA resulted in reduced expression of CDK5R1 protein, after 20 nM (Fig. 1i). Thus, the abovementioned results clearly showed that miR-152 targeted CDK5R1 and reduced its protein expression.

### Cleavage of CDK5R1 (p35 into p25) activate CDK5

**Figure 2:**
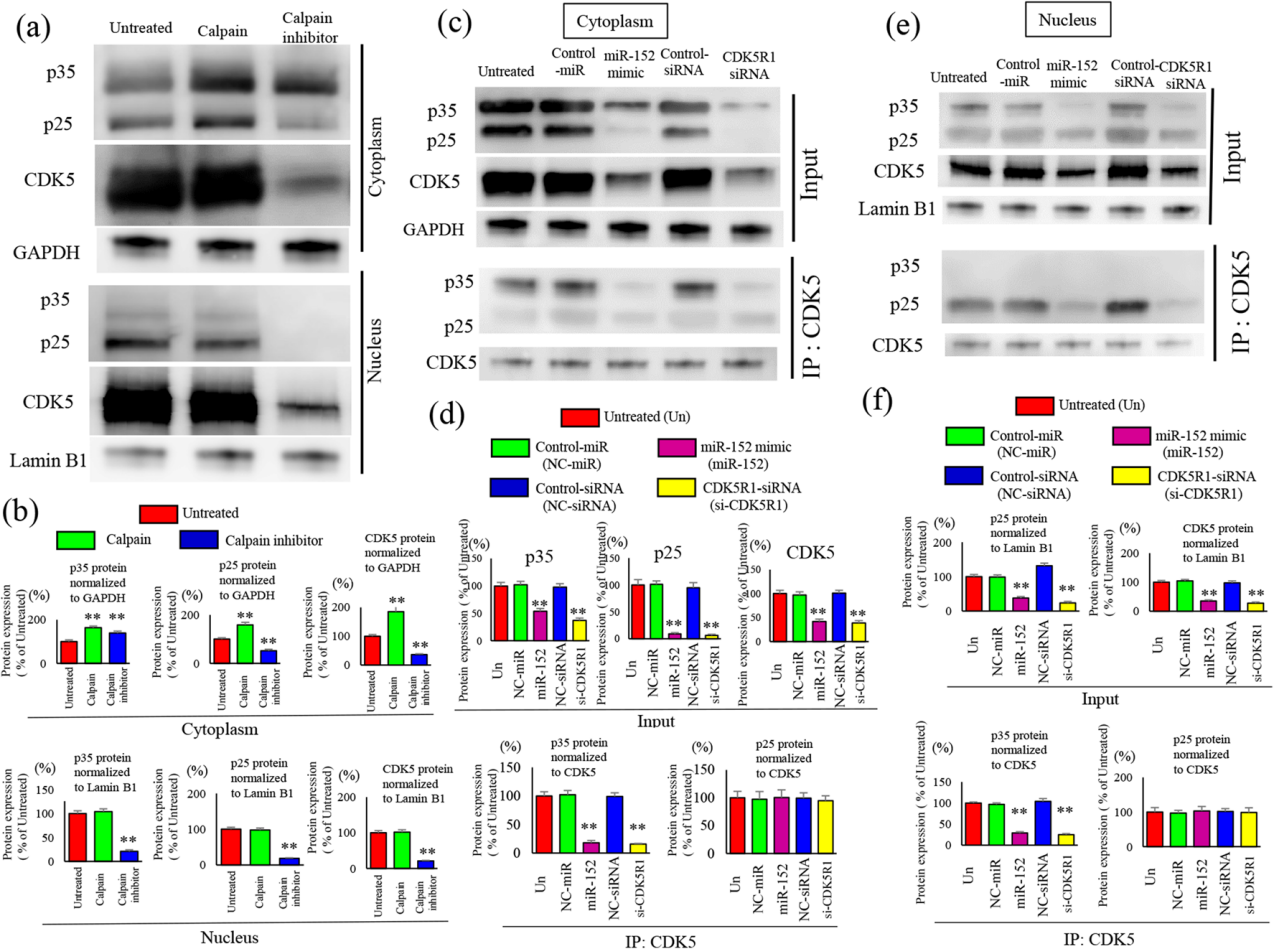
Changes in the expression of p35/p25 in the cytoplasm and nucleus, and changes in the expression of CDK5R1 after transfection with miR-152. Effects of activation and inhibition of calpain on p35/p25, and subsequent changes in CDK5 activity in the cytoplasm and nuclear fractions were analyzed by immunoblotting (**a**) and protein expression quantification (**b**). The cytoplasmic fraction was normalized using GAPDH, and the nuclear fraction was normalized using Lamin B1 as reference. In the nuclear fraction, p25 was increased by calpain activation and p35 was absent, whereas nuclear p25 was decreased by calpain inhibitor. Cytoplasmic p25 was formed from the cleavage of p35 by calpain activation and inhibition. Effects of miR-152 mimic transfection and CDK5R1 knockdown on p35/p25 and CDK5 in the cytoplasm fractions were analyzed by western blot analysis (**c**) and band concentration quantification (**d**). Immunoprecipitation (IP) was performed with CDK-5, followed by western blotting with the indicated antibodies. The Input was normalized using GAPDH, and the proteins immunoprecipitated with CDK5 were normalized to CDK5. Western blotting analysis of p35/p25 levels in SKES-1 cells transfected with miR-152 and CDK5R1 siRNA in nucleus fraction (**e**). Immunoprecipitation (IP) was performed with CDK-5, followed by western blotting with the indicated antibodies. Quantification (**f**) of changes in the protein expression of co-precipitated p35/p25 in the nuclear fractions were performed. The Input of nucleus fraction was normalized using Lamin B1, and the proteins immunoprecipitated with CDK5 were normalized to CDK5.

Cleavage of CDK5R1 (p35) into p25 by calpain in the cytoplasm leads to the formation of the CDK5-p25 complex, which is then translocated into the nucleus where it is activated and increases the kinase activity^16^. To determine whether this mechanism can also be replicated in SKES-1 cells, we evaluated the dynamics of p35/p25 and CDK5 in the cytoplasm and nuclear fractions after calpain activation and calpain inhibitor administration (Fig. 2a). In the cytosolic fraction, activation of calpain led to the cleavage of p35 into p25, which was then blocked by a calpain inhibitor. Overexpression of p25 led to the activation of CDK5, which showed that calpain cleaved p35 into p25 while inducing CDK5 overexpression in SKES-1 cell lines as well. In the nuclear fraction, calpain activation led to overexpression of p25 and absence of p35, and nuclear p25 was downregulated by a calpain inhibitor. Calpain activation and inhibition resulted in the cleavage of p35 to form p25. In SKES-1 cells, p35 was cleaved in the cytoplasm to form p25, which was subsequently translocated to the nucleus, indicating that CDK5 was activated (Fig. 2b).

Next, we transfected miR-152 or CDK5R1 siRNA to evaluate changes in the expression of p35 and subsequent changes in the activity of CDK5 (and its cofactor, p25) as a complex (Fig. 2c). We observed reduced expression of p35 and p25 and CDK5 in the cytosolic fraction (input) in the miR-152 mimic and CDK5R1-siRNA transfection group. Further, IP using CDK5 antibodies showed reduced expression of p35 in the cytosolic fraction in miR-152 mimic and CDKR1-siRNA groups (Fig. 2d). The expression of p35/p25 and CDK5 in the nuclear fraction was significantly reduced in the miR-152-CDK5R1-siRNA transfection group compared with the untreated group (Fig. 2e).

In the nuclear fraction, input showed a reduction in p35 expression in the miR-152 mimic and siRNA treatment groups, and IP with the CDK5 antibody showed a reduction in p25 expression (Fig. 2f). Our results demonstrated that reduced expression of miR-152 in all ES cells allowed the overexpression of p35/p25, which led to the activation of CDK5 in the nucleus.

### miR-152 suppressed cell proliferation due to cell cycle retardation

**Figure 3:**
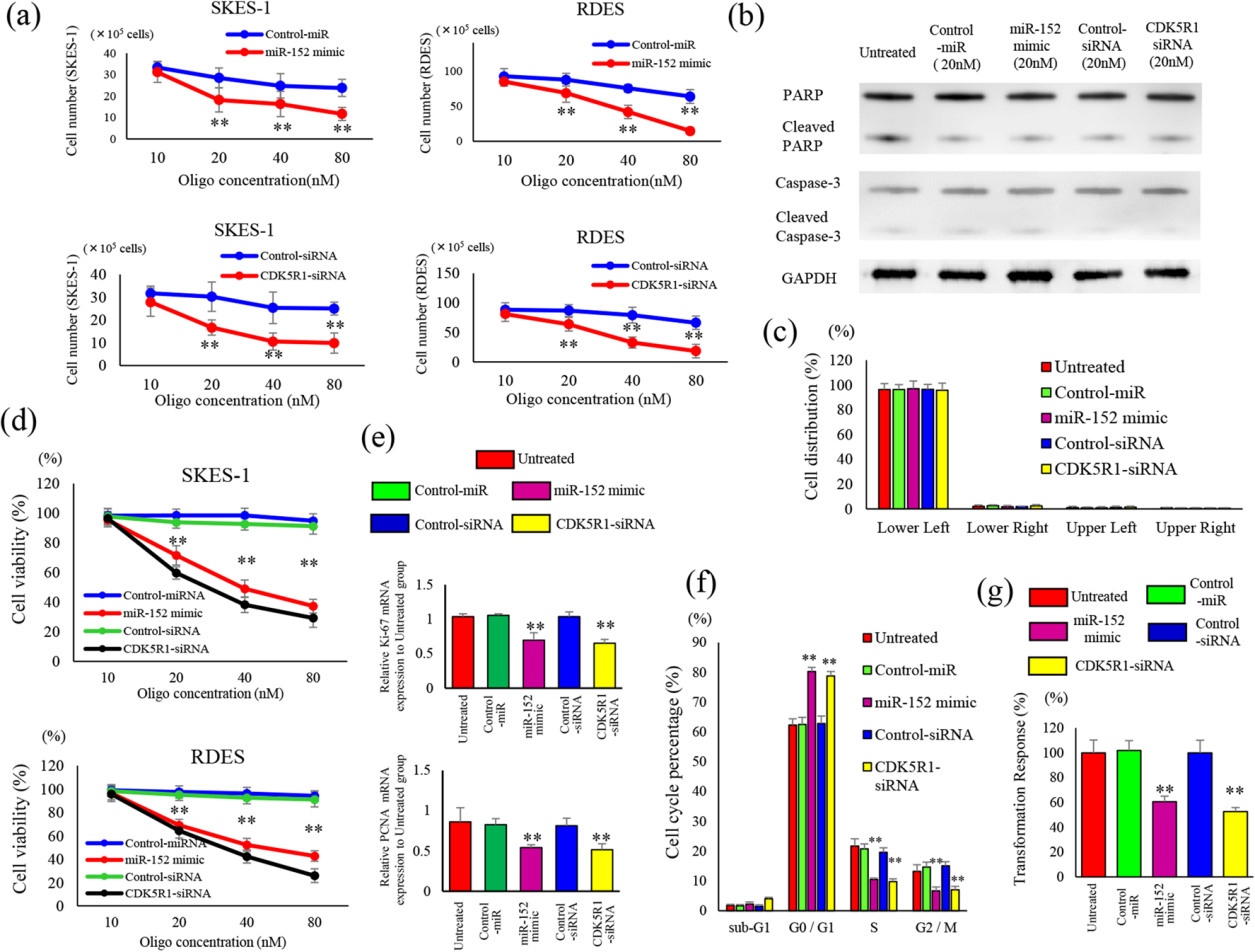
Effects and causes of changes in miR-152 and CDK5R1 expression on cell proliferation. (**a**) Cell proliferation of SKES-1 and RDES cells transfected with miR-152 mimic or CDK5R1 siRNA were compared with those in the negative control group. Data represent mean ± SEM of three independent experiments. SKES-1 cells transfected negative control-miR (20 nM), miR-152 mimic (20 nM), negative control-siRNA (20 nM), CDK5R1-siRNA (20 nM) and untreated cells, followed by western blot analysis with the indicated antibodies. Images (**b**) from immunoblot analysis of apoptosis induction after transfection with miR-152 mimic and CDK5R1 siRNA. GAPDH level was used as loading control. (**c**) Ewing’s sarcoma cells were stained with Annexin V-FITC/PI to assess cell apoptosis. Each quadrants represent viable cells (Lower Left quadrant), early apoptotic cells (Lower Right), late or secondary necrotic cells (Upper Right), and primary necrotic cells (Upper Left), respectively. (**d**) The viability of SKES-1 and RDES cells was determined using Cell Counting Kit‑8 assays. (**e**) Cell proliferation was assessed by observing the changes in the expression of Ki-67 and PCNA using qPCR. SKES-1 cell-transfected negative control-miR (20 nM), miR-152 mimic (20 nM), negative control-siRNA (20 nM), CDK5R1-siRNA (20 nM) and untreated cells, followed by flow cytometric analysis with 5-bromo-2 deoxyuridine-7-amino-actinomycin D (BrdU-7-AAD) staining. Changes in cell cycle for each condition were analyzed by the BrdU-7-AAD double staining assay (Supplemental Fig.S3c) and the rate of cell-cycle progression (**f**). (**g**) Effect of miR-152 mimic and CDK5R1 knockdown on the viability of SKES-1 cells for their ability to grow in soft agar. The results are represented as the mean ± SEM (n.s., no significance). **p < 0.01 versus the related control groups.

We examined the effect of transfection with miR-152 mimic and CDK5R1 siRNA on cell proliferation. When compared to the control group, the proliferation of SKES1 cells was separately inhibited by 20 nM of miR-152 mimic and CDK5R1 siRNA. Additionally, growth changes were observed in RDES cells. Proliferation assays in SKES-1 and RDES showed a significant decrease in the miR-152 mimic (20 nM) and CDK5R1 siRNA-treated group when compared to the untreated group (Fig. 3a). An apoptosis assay was performed to examine the possible cytostatic effects caused by the induction of apoptosis (Fig. 3b). Expression of cleaved PARP and cleaved caspase 3 were unaltered in the miR-152-CDK5R1-siRNA group compared with that in the untreated group, whereas transfection with miR-152 or CDK5R1 siRNA reduced the expression of CDK5R1, thereby leading to reduced cell proliferation (Supplemental Fig. S3a). However, the induction of apoptosis did not contribute to this process at the condition in which growth inhibition was observed in the proliferation assay. Furthermore, flow cytometry analysis using Annexin V-FITC/PI double staining showed that cell apoptosis was not observed among all groups (Supplemental Fig. S3b). Treatment with miR-152 mimic and CDK5R1 knockdown did not induce apoptosis (Fig. 3c). CCK‑8 assays showed a decrease in cell viability following transfection with miR‑152 mimic and CDK5R1 knockdown (Fig. 3d). miR-152 induction and CDK5R1 knockdown decreased the mRNA expression of Ki-67 and PCNA, indicating a reduction in cell proliferation ability (Fig. 3e). Next, a cell cycle analysis of BrdU uptake was performed (Supplemental Fig. S3c). Acceleration of the cell cycle in the G1 phase and delay in the cell cycle in the S and G2/M phases were observed in the miR-152 mimic and CDK5R1 siRNA groups compared with the untreated group (Fig. 3f). The delay in the cell cycle, and not cell death by apoptosis, caused a reduction in cell proliferation after transfection with miR-152 and knockdown of CDK5R1. The colony‑formation assays indicated a considerable decrease in colony formation in SKES-1 cells following transfection with miR-152 mimic and CDK5R1 knockdown (Supplemental Fig. S3d). A statistically significant reduction of the transformation response in the presence of miR-152 mimic and CDK5R1 knockdown in comparison with both untreated and control groups (Fig. 3g).

### Changes in the expression of cell cycle factors in each phase

**Figure 4:**
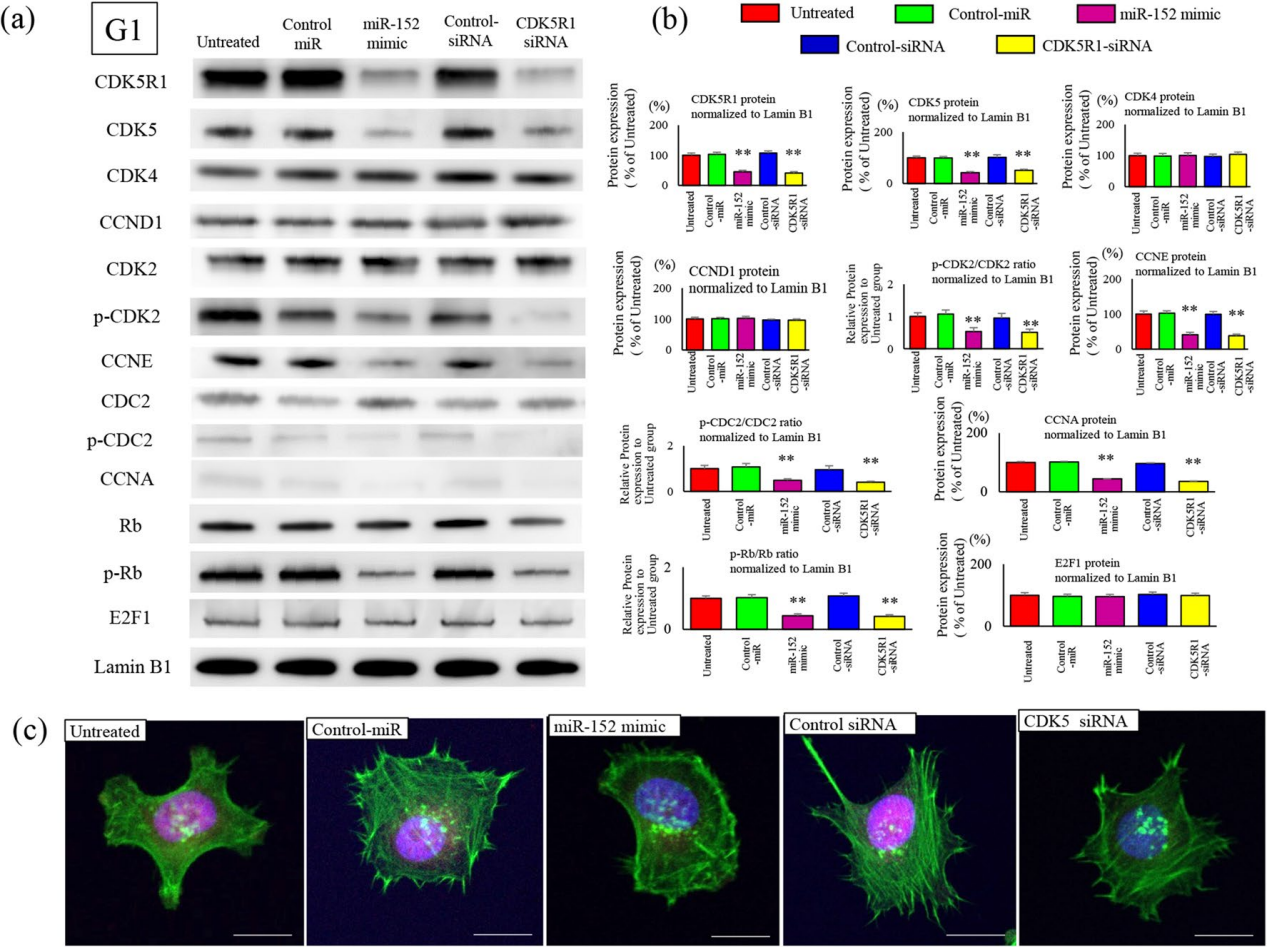
Effects of cell cycle factors after transfection with oligonucleotides in the G1 phase. Negative control-miR (20 nM), miR-152 mimic (20 nM), negative-control siRNA (20 nM), and CDK5R1-siRNA (20 nM) were transfected in SKES-1 cells and were synchronized at different stages of the cell cycle: G1 phase, thymidine treatment (2 mM, 24 h). Nuclear protein was extracted, and western blotting was performed with the indicated antibodies. Phosphorylation of p-CDK2 (Thr160), p-CDC2 (Thr161), and p-Rb (Ser807/811) is inhibited by treatment with miR-152 mimic and CDK5R1 siRNA. Cell cycle factors after treatment were analyzed by immunoblotting (**a**) and band concentration quantification (**b**). The protein bands of Cell cycle‑related proteins were quantified and normalized to Lamin B1. The results are represented as the mean ± SEM (*n*.*s*. no significance). **p < 0.01 versus the related control groups. C. Representative confocal images of Phosphorylated (p) -Rb are shown. p-Rb was stained red; whereas, F-actin was stained with phalloidin in green (**c**). Nuclear staining was performed with DAPI. Scale bar, 20 μm.

**Figure 5:**
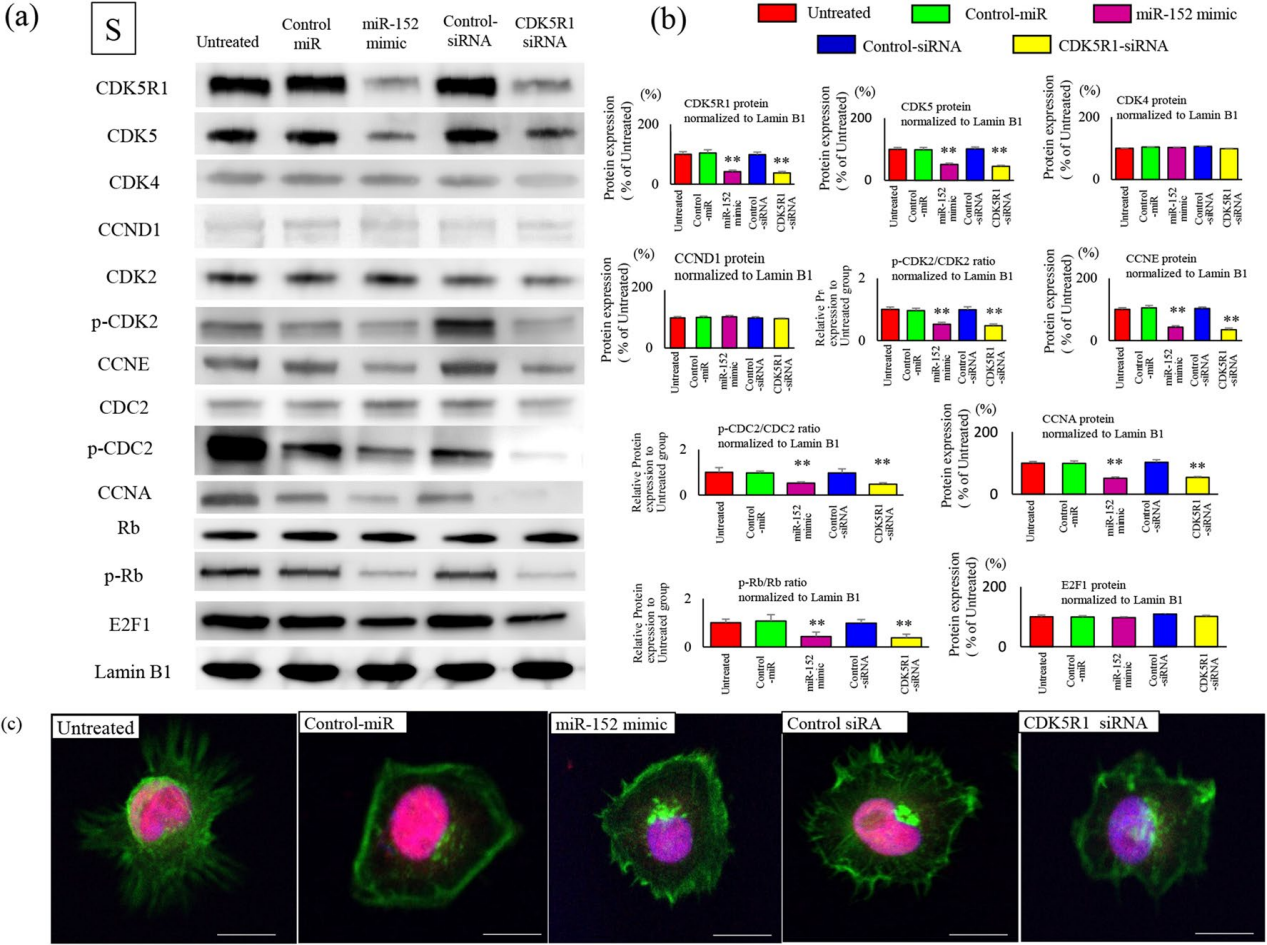
Effects of cell cycle factors after transfection with oligonucleotides in the S phase. Negative control-miR (20 nM), miR-152 mimic (20 nM), negative-control siRNA (20 nM), and CDK5R1-siRNA (20 nM) were transfected in SKES-1 cells and were synchronized at different stages of the cell cycle: S phase, hydroxyurea treatment (3 mM, 24 h). Nuclear protein was extracted, and western blotting was performed with the indicated antibodies. Cell cycle factors after treatment were analyzed by immunoblotting (**a**) and band concentration quantification (**b**). The protein bands of Cell cycle‑related proteins were quantified and normalized to Lamin B1. The results are represented as the means ± SEM (*n*.*s*. no significance). **p < 0.01 versus the related control groups. C. Representative confocal images of p-Rb are shown. p-Rb was stained red, whereas F-actin was stained with phalloidin in green (**c**). Nuclear staining was performed with DAPI. Scale bar, 20 μm.

**Figure 6:**
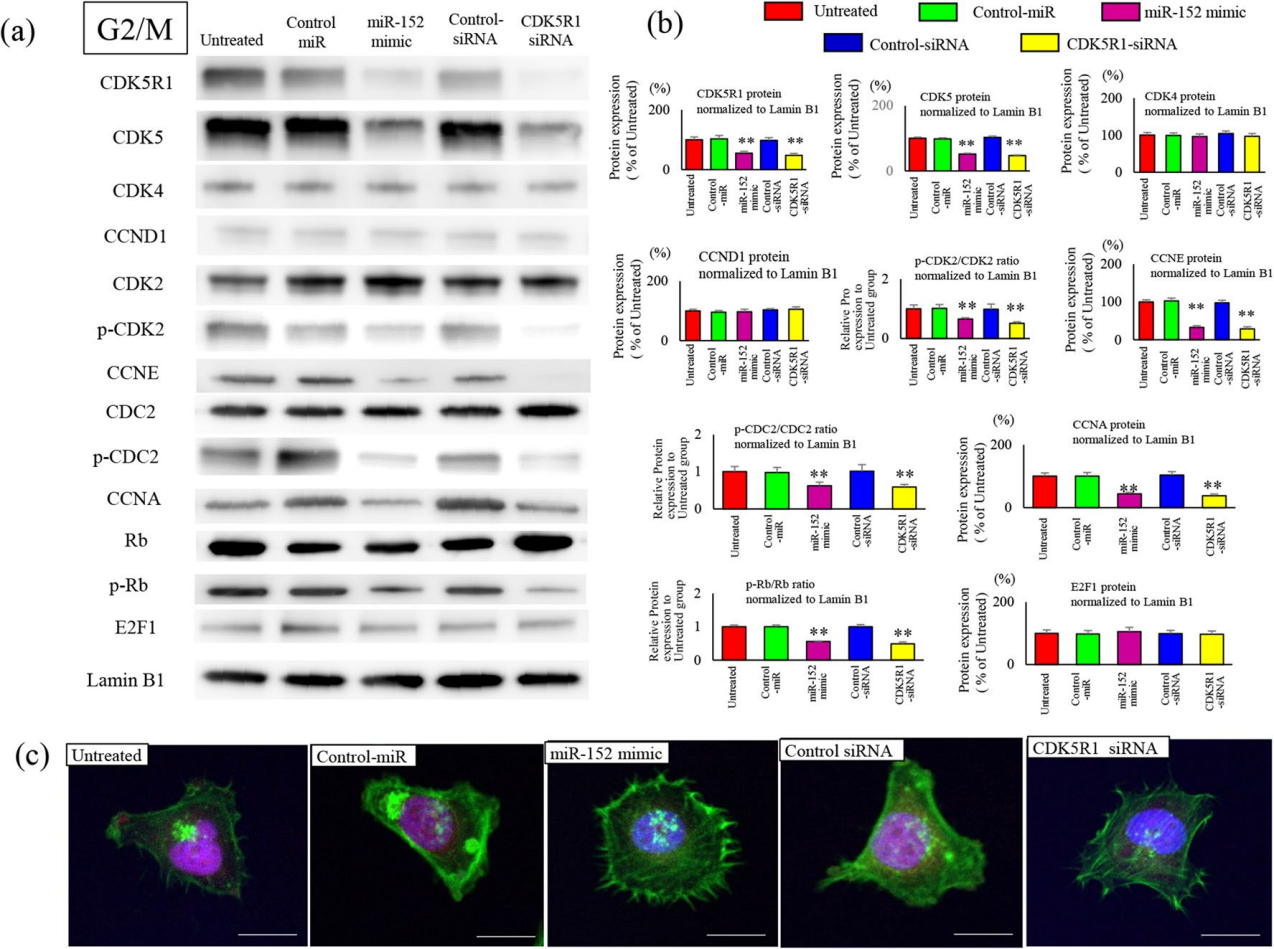
Effects of cell cycle factors after transfection with oligonucleotides in the G2/M phase. Negative control-miR (20 nM), miR-152 mimic (20 nM), negative-control siRNA (20 nM), and CDK5R1-siRNA (20 nM) were transfected in SKES-1 cells and were synchronized at different stages of the cell cycle: G2/M phase, nocodazole treatment (50 ng/mL, 24 h). Nuclear protein was extracted, and western blotting was performed with the indicated antibodies. Cell cycle factors after treatment were analyzed by immunoblotting (**a**) and band concentration quantification (**b**). The protein bands of Cell cycle‑related proteins were quantified and normalized to Lamin B1. The results are represented as the mean ± SEM (*n*.*s*. no significance). **p < 0.01 versus the related control groups. (**c**) Representative confocal images of p-Rb are shown. p-Rb was stained red, whereas F-actin was stained with phalloidin in green. Nuclear staining was performed with DAPI. Scale bar, 20 μm.

To examine the effects of miR-152 and CDK5R1 on the cell cycle, changes in cell cycle factors were synchronized in the G0/G1, S, and G2/M phases. Expression of phosphorylated Rb (p-Rb) in the G1 phase after transfection with miR-152 mimic or CDK5R1 siRNA was significantly reduced compared with that in the untreated miR-control and siRNA-control groups (Fig. 4a). Expression of CCNE was significantly reduced in the miR-152 transfection and CDK5R1 knockdown group compared with the untreated miR-control and siRNA-control groups. Consistent with the decrease in CCNE expression, p-CDK2 (Thr160) and p-CDC2 (Thr161) expression was also significantly reduced. CDK2 is actively phosphorylated and downstream Rb is phosphorylated (Ser807/811) due to higher CCNE in ES cells than in normal cells. Thus, miR-152 transduction and CDK5R1 reduction appropriately suppressed CDK2 and Rb activity (Fig. 4b). Immunofluorescence staining showed that nuclear expression of p-Rb was significantly reduced in the miR-152-transfection-CDK5R1-knockdown group compared with the untreated groups. Compared with the restriction point (R-point), the expression level of CCNE is a more significant contributor of timing the cell division, which is strictly controlled in the G1 phase (Fig. 4c). In the S phase, CCNE overexpression occurred upon increased Rb phosphorylation, even in normal cells. Both CCNA expression and CDC2 phosphorylation occurred actively; the expression of CDK2 and Rb were also reduced by miR-152 transfection and CDK5R1 knockdown (Fig. 5a). The expression of p-Rb was significantly reduced in cells transfected with miR-152 and CDK5R1 knockdown compared with cells of the untreated cells (Fig. 5b). The phosphorylation of RB and nuclear accumulation after immunofluorescence staining were significantly reduced in cells with miR-152 transfection and CDK5R1 knockdown compared with untreated cells (Fig. 5c). In the G2/M phase, the levels of CCNE and CCNA, which should be reduced, remain unchanged, and the phosphorylation of CDK2 and CDC2 is high (Fig. 6a). In ES cells, the phosphorylation of Rb continues in the G2/M phase along with the expression of target gene. These are suppressed by miR-152 transfection and CDK5R1 knockdown (Fig. 6b). Reduced expression of Rb protein phosphorylation was observed in cells with miR-152 transfection and CDK5R1 knockdown (Fig. 6c). This indicates the overexpression of CCNE in cells with reduced expression of miR-152 and overexpression of CDK5R1, even in the G2/M phase. Phosphorylation of Rb proteins in the initial G1 phase after cell division and subsequent overexpression of CCNE after cell division created conditions that allowed cells to easily cross the R-point.

### Upregulated miR-152 reduced tumor size in mice and decreased CCNE expression in tumor tissues

**Figure 7:**
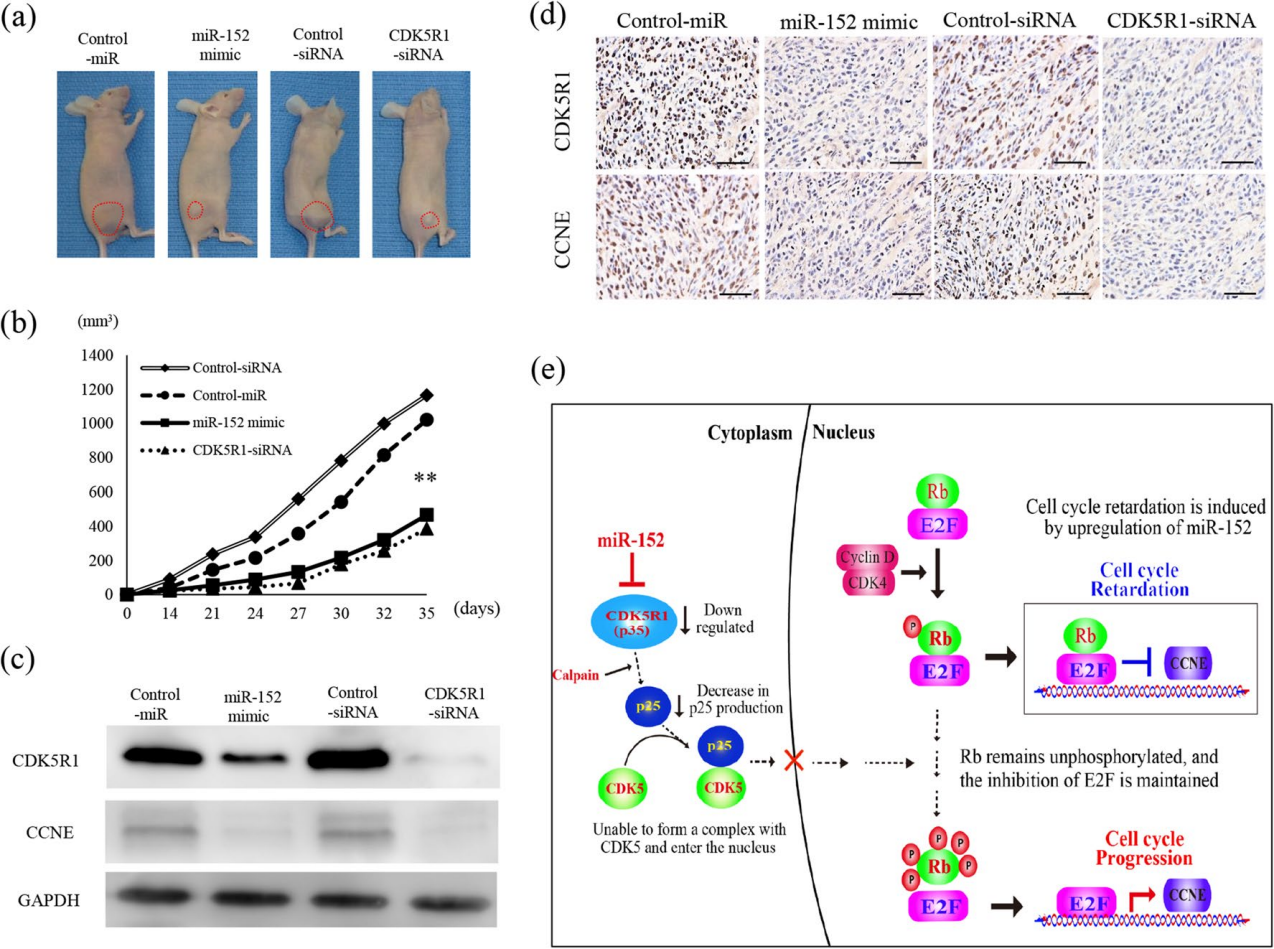
Effects of miR-152 and CDK5R1 were examined in mice. SKES-1 cells (1 × 10^6^) were inoculated into the gluteal region of BALB/c nu/nu mice. After 5 weeks, the mouse tumors were collected and analyzed for further experiments. Tumor volumes in implanted mice (**a**) were compared in each group (**b**). The subcutaneous tumors are indicated by red dashed lines. The collected mouse tumor tissues were analyzed by protein expression (**c**) and immunostaining of CDK5R1 and CCNE (**d**) per quantification unit area in CDK5R1- and CCNE-positive cells was quantified. The densitometric levels of protein bands were quantified and normalized to that of GAPDH. Original magnification,  × 400; Scale bars: 50 μm. Data represent mean ± SEM of three independent experiments. **p < 0.01 versus the related control groups. (**e**) The schema illustrates that low expression of miR-152 causes accumulation of CDK5R1 and contributes to high expression of CCNE.

Tumors from mice xenografted with Ewing’s sarcoma cells: Control-miR, miR-152-mimic, Control-siRNA, and CDK5R1-siRNA-transfected cells, were analyzed (Fig. 7a).

Significant tumor shrinkage was observed in the miR-152 mimic-CDK5R1 knockdown groups compared with the negative control groups (Fig. 7b). CDK5R1 and CCNE protein levels were significantly downregulated in tumor tissues collected from the miR-152-mimic and CDK5R1-siRNA groups (Fig. 7c and Supplemental Fig. S4a). Expression of CDK5R1 and CCNE in tumor tissues was evaluated by immunostaining (Fig. 7d). The number of CDK5R1-positive cells was significantly lower in the miR-152 and CDK5R1 knockdown group compared with the negative control group. Similarly, the number of CCNE-positive cells was significantly lower in the miR-152-CDK5R1-knockdown group than in the negative control groups (Supplemental Fig. S4b). In Ewing sarcoma cells, miR-152 was down-regulated and its target, CDK5R1, was up-regulated. Within the cytoplasm, p25 underwent cleavage by calpain, was bound to cytoplasmic CDK5, and was translocated to the nucleus. Rb protein was phosphorylated by the activated CDK5, and CCNE expression was upregulated, thus leading to CCNA accumulating as a positive loop and resulting in abnormal cell-cycle progression (Fig. 7e).

## Discussion

ES is a highly malignant sarcoma that is commonly observed in young adults and mainly caused by the fusion gene EWSR1-Fli1, which is a member of the ETS family^17^. In this study, we demonstrated the relationship between abnormalities in the expression of miR-152, one of the tumors suppressive miR, and its target gene CDK5R1 in ES. Our previous findings suggested the involvement of CCNE in the proliferation of ES cells^18^, and whole genome analysis of the cDNA array and cell cycle-related factors demonstrated the overexpression of CDK5R1 in all ES cell lines, thereby aiding in elucidating the underlying mechanism.

CDK5R1, known as p35, activates CDK5 serine/threonine kinase by binding to CDK5^19^. Calpain protein cleaves p35 into p25, forming a complex with CDK5, which is then translocated into the nucleus to promote cell proliferation through Rb phosphorylation^20^. CDK5, which is mainly expressed in nerve cells, plays an important role in neural tissues (e.g. controlling the function of the central nervous system, synaptogenesis, etc.)^21, 22^ and is involved in cell proliferation through CDK5 upregulation in malignant cells^10^. We demonstrated that miR-152 targeted CDK5R1 at the mRNA and protein expression levels. Further, we showed that following reduced miR-152 expression, calpain cleaved the CDK5-p35 complex into CDK5-p25, which was then translocated into the nucleus and activated; this is consistent with previous studies. Calpain stimulation did not induce changes in nuclear p25 levels significantly. It is possible that in Ewing sarcoma cell lines, the persistent high expression of CDK5R1 leads to a state of high endogenous p25 production. Therefore, even with external Calpain stimulation, it may not have resulted in a more significant difference. On the contrary, the decrease in nuclear p25 upon Calpain inhibition suggests the validation of the cleavage of p35 into p25 by Calpain. The reduced expression of CDK5R1 after transfection with miR-152 mimic or CDK5R1 siRNA showed cytostatic effects via a delay in the cell cycle. Previous studies have also reported CDK5-mediated upregulation of the cell cycle and tumorigenesis^9^.

Due to the reduced expression of CDK5R1, reduced phosphorylation of Rb proteins and reduced expression of E2F1 and CCNE in the G1 phase. In the G1 phase, E2F1 is usually inhibited by Rb if the cells pass through the R-point without phosphorylation of Rb proteins^23^. In ES cells, overexpression of cellular CCNE from the G1 phase did not affect the expression of CCNE, thereby suggesting that CCNE overexpression was possibly induced by the phosphorylation of Rb proteins.

miR-152 transfection and CDK5R1 knockdown (CDK5R1 siRNA-transfected) led to the downregulation of Rb phosphorylation and reduced expression of CCNE and CCNA. Reduced Rb phosphorylation, CCNE and CCNA expression after reduced expression of CDK5R1 were observed in the S phase as well. Interestingly, the expression of CCNE persisted in the G2/M phase, instead of being reduced. Throughout the cell cycle, phosphorylation of Rb proteins through CDK5 suggests the direct phosphorylation of Rb proteins mediated by CDK5. Further, we revealed miR-152 to be a tumor-suppressive miRNA that reduces the expression of p35 and inhibits the activation of CDK5-p25 complex in the nucleus of ES cells. The results indicate that a stable supply of CDK5 cleaved from CDK5R1 to the nucleus, regardless of the cell cycle phase, causes phosphorylation of Rb. This suggests that E2F1 permanently expresses its target genes, CCNE and CCNA, which is consistent with our results.

The finding that reduced expression of CDK5R1 led to tumor shrinkage in mice is consistent with previous findings on the cell proliferative effect of CDK5R1. It was shown that the transfection with miR-152 mimic reduced the expression of CDK5R1 and CCNE in tumor tissues, even in mice. Reduced expression of miR-152 that was observed in all ES cells allowed activation of CDK5 in the nucleus via the overexpression of p35 and p25, thereby indicating a relationship between the reduced expression of tumor suppressive miR-152 and continuous activation of CCNE through various factors. Previous studies have reported the overexpression of CCNE in cancer cells as well.

Our current findings on the activation of CDK5 induced by the overexpression of CDK5R1 and p25 are consistent with those reported mainly in neuronal studies. However, the effects of CDK5 have not been previously discussed in detail, and this study elucidated the mechanism by which abnormal control by miRNAs disrupted CCNE expression via cell cycle progression through Rb-related disinhibition. The relationship between CDK5R1 and Rb phosphorylation and CCNE/CDK2 in ES has not been fully elucidated. However, CCNE has been identified as a predictor of therapeutic efficacy because its overexpression reduces sensitivity to CDK4/6 inhibitors^24^. Drugs targeting the cell cycle through Rb phosphorylation are currently in clinical use, and CCNE is the final effector molecule. Thus, our results demonstrate the utility of our approach for the development of effective individualized treatments for ES.

## Materials and methods

### Ethical approval

Each author certifies that his or her institution has approved the animal protocol for this investigation and that all investigations were conducted in conformity with ethical principles of research. Mouse experiments were approved by the Medical Ethics Committee of Oita University (No. 182403) and all experiments were performed in accordance with relevant guidelines and regulations. All animal experimental procedures were performed in accordance with ARRIVE guidelines^25^.

### Cell lines

ES cell lines, SKES-1, RDES, SKNMC, and SCCH were purchased from the Japanese Collection of Research Bioresources Cell Bank (Tokyo, Japan), and the WE68 cell line was kindly provided by Dr. Frans van Valen (Westfalische-Wilhelms University, Munster, Germany). Human mesenchymal stem cells (hMSCs) were obtained from TaKaRa Biotechnology (Otsu, Japan). All cell lines used are routinely (every 3 months) tested for Mycoplasma using the e-Myco PLUS Mycoplasma PCR Detection Kit (LiliF Diagnostics). RDES and SKNMC cells were cultured in Dulbecco’s modified eagle medium (DMEM) high glucose medium (Invitrogen, NY) with 10% FBS and 1% penicilium and streptomycin. SKES1 cell and WE-68 cells were cultured in RPMI 1640 (Invitrogen, NY) supplemented with 10% FBS. SCCH cells were grown in minimal essential medium (MEM) supplemented with 10% fetal bovine serum (FBS; Invitrogen, NY) and 0.1 mmol/L nonessential amino acids (NEAA). hMSCs were cultured with the Chemically Defined Mesenchymal Stem Cell Basal Medium (MSCBM-CD) with MSCGM-CD SingleQuats (TaKaRa Biotechnology). Cells were expanded for 8 to 9 days and no later than 4 to 5 passages after thawing. The cells were incubated at 37 ºC in an incubator chamber supplemented with 5% CO_2_ and passaged when the cells were grown to approximately 70% confluent, as previously described^26^. Mycoplasma contamination was tested monthly using the MycoAlertTM Mycoplasma kit (Lonza). Cells were not authenticated in-house and were not passaged more than 30 times.

### Genome-wide miRNA expression microarray and cDNA arrays

GeneChip miRNA 3.0 array (Affymetrix, Santa Clara, CA, USA) was used for miRNA, and GeneChip Genome HG U133 Plus 2.0 Array (Affymetrix) was used for mRNA expression profiling in all five ES cell lines and hMSCs, as previously described^27^. The gene list was filtered with a fold-change cutoff of 2, i.e. we obtained a list of genes with significant differential expression of two folds or more.

### Reverse transcriptase quantitative polymerase chain reaction (RT-qPCR)

Total RNA extraction and RT-qPCR were performed as previously described^28^. The primer sequences used for qPCR were as follows: CDK5R1-forward 5′-TGCAATGGTGACCTCGATCC-3′ and CDK5R1-reverse 5′-AAGGTTCTGCAGCAGATCCC-3′, Ki-67 forward: 5′- GAAAGAGTGGCAACCTGCCTTC -3′, Ki-67 reverse: 5′- GCACCAAGTTTTACTACATCTGCC -3′, PCNA forward: 5′- CAAGTAATGTCGATAAAGAGGAGG -3′, PCNA reverse: 5′- GTGTCACCGTTGAAGAGAGTGG -3′. GAPDH-forward 5′-CCACAGTCCATGCCATCACT-3′ and GAPDH-reverse 5′-GAGATTCAGTGTGGTGGGGG-3′.

### Analysis to confirm whether miR-152 targeted CDK5R1 mRNA

We confirmed the expression of CDK5R1 mRNA by transfection with oligonucleotides (miR-152 mimic [5′-CAGUGCAAUAGUAUUGUCAAAGC-3′] and miR-152 mutant [5′-CUCACGUAUAGUAUUGUCAAAGC-3′]), which were developed by preventing gene expression and using the method described in our previous study^29^. In addition, we used a luciferase assay to examine whether miR-152 directly targeted CDK5R1 mRNA. The 3′-untranslated region (3′-UTR) of CDK5R1 was cloned into the pmirGLO plasmid, and luciferase activity assay was performed using the Dual-Luciferase® Reporter assay kit (Promega Corp., Madison, WI, USA) according to the manufacturer’s instructions. SKES-1 cells were plated in 96-well luminescence plates and conventional 96-well cell culture plates prior to transfection and cultured to 70% confluence for 24 h. Then, the cells were co-transfected for 6 h with pmirGLO‑CDK5R1‑3′-UTR luciferase plasmid and either the control-miR or miR-152 using Lipofectamine™ 2000 reagents, and then incubated for 24 h. Transfected cells were lysed using a buffer-substrate solution. After 30 min of incubation, luciferase activity of each well was detected using a Dual‑Luciferase Reporter Assay System (E1910; Promega).

### Immunoprecipitation (IP)

Cells cultured in 6-well plates were harvested and solubilized in lysis buffer (Pierce IP lysis buffer; Thermo Fisher Scientific, Inc., Waltham, MA, USA). After centrifugation, the supernatant was incubated for 12 h at 4 °C with 2 mg of anti-CDK5 (#12134; Cell Signaling Technology, Tokyo, Japan). Following the addition of 30 mL Protein G Sepharose™ 4 beads (GE Healthcare, Chicago, IL, USA), the mixture was incubated for 2 h at 4 °C with rotation. Immune complexes were washed three times with lysis buffer, and the Sepharose beads were boiled for 10 min in sample buffer. Immunoprecipitates were run on sodium dodecyl sulphate–polyacrylamide gel electrophoresis (SDS-PAGE) using 4–20% gradient precast gels (Bio-Rad), followed by western blot analysis with primary antibodies against p35/p25 (#2680, Cell Signaling Technology) and CDK5.

### Oligonucleotide transfection and proliferation assay

Oligonucleotide transfection and cell proliferation assays were performed as previously described^30^. Briefly, transfection with miR-152 mimic (10, 20, 40, 80, and 160 nM), negative control miRNAs (control-miR; 20 nM), CDK5R1 siRNA (10, 20, 40, 80, and 160 nM), and negative control-siRNA (control-siRNA; 20 nM; Invitrogen, Thermo Fisher Scientific) was performed using Lipofectamine 2000 reagent (Invitrogen) in antibiotics-free OptiMEM (Invitrogen) according to the manufacturer’s instructions. After 48 h of cultivation, the cells were counted using a TC10 Automated Cell Counter (Bio-Rad Laboratories, Inc., Hercules, CA, USA).

### Cell cycle analysis

A 5-bromo-2-deoxyuridine (BrdU) incorporation assay was conducted to monitor DNA replication using a fluorescein isothiocyanate (FITC) BrdU Flow Kit (BD Bioscience, Bedford, MA, USA) according to the manufacturer’s instructions. Briefly, cells were synchronized by starvation in Dulbecco’s Modified Eagle’s Medium supplemented with 1% fetal bovine serum (FBS) for 48 h before treatment with 10% FBS and 100 µM BrdU for 6 h. SKES-1 cells were fixed and permeabilized with BD Cytofx/Cytoperm Buffer, BD Buffer Plus, and BD Cytofx/Cytoperm Buffer in sequential order. SKES-1 cells were treated with deoxyribonuclease before incubation with an anti-BrdU-FITC antibody and 7-amino-actinomycin D (7-AAD). The samples were analyzed by FACS Fortessa using FACSuite and Flow Jo analysis software (BD Bioscience). The percentage of cells in G0/G1, S, and G2/M phases were counted and compared.

### Apoptosis assay

Apoptotic cell death was detected by western blot analysis using antibodies against PAR/poly (ADP-ribose) polymerase (PARP; #9542), cleaved PARP (#9541), caspase 3 (#9662), cleaved caspase 3 (#9661), all of which were purchased from Cell Signaling Technology. Additionally, apoptotic cell death was determined by FACS using an Annexin V-FITC apoptosis detection kit (BD Biosciences). Briefly, cells were incubated with the staining solution in Annexin V-FITC/PI and FITC and PI fluorescence were detected at 488 nm and 530 nm emission wavelength using a flow cytometer.

### Cell counting kit‑8 (CCK‑8) assay

Cell viability assay was performed using CCK‑8 kit (Abcam, cat. no. ab228554). Briefly, cells were seeded into 96‑well microtiter plates at a density of 6.0 × 10^3^ cells/well, followed by transfection with 10, 20, 40, and 80 μM of miR-152 mimic and the addition of 10 µL of CCK‑8 solution to each well. After incubation for 2 h, the absorbance was measured at 450 nm using a spectrophotometer. Cell viability was calculated based on the absorbance values obtained in the experimental group and the control group.

### Transformation in soft agar

Anchorage-independent transformation assays were performed using a CytoSelect 96-well cell transformation assay kit (Cell Biolabs, Inc.). Briefly, cells were plated in soft agar in a 96-well plate at 5000 cells/well and cultured for 5 days. The transformation was performed according to the manufacturer’s protocol.

### Cell cycle synchronization

SKES-1 cells were synchronized at different stages of the cell cycle: G1 phase, thymidine treatment (2 mM, 24 h); S phase, hydroxyurea treatment (3 mM, 24 h); and G2/M phase, nocodazole treatment (50 ng/mL, 24 h) in accordance with a modified protocol.

### Western blot analysis

Western blot analysis was adopted from a previous study^27^. Peroxidase-conjugated anti-rabbit IgG secondary antibodies (Jackson ImmunoResearch Laboratories Inc., West Grove, PA, USA) were used at a 1:2000 dilution.

### Immunohistochemistry and immunofluorescence

Immunostaining for CCNE (ab74276; Abcam, Cambridge, MA, USA), Phospho-Rb (Ser807/811) Antibody (#9308, CST) and CDK5R1 (ab235271; Abcam) was performed as previously described^30^. A rabbit anti-CCNE antibody was used for immunofluorescence staining. Following three washes with phosphate-buffered saline, the cells were incubated with a goat anti-rabbit Alexa Fluor 594 antibody (Thermo Fisher Scientific) and Alexa Fluor® 488 Phalloidin Conjugate (A12379, Thermo Fisher Scientific). Then, the mounting medium with 4′,6-diamidino-2-phenylindole (DAPI; Vector Laboratories, Inc., Burlingame, CA, USA) was applied to the cells, and the cells were fixed in a dark room for 6 h. Confocal z-stack images were acquired using a Zeiss 710 confocal microscope and ZEN software package (Carl Zeiss, Oberkochen, Germany).

### Animal experiments

All animal experiments were conducted in accordance with the accepted standards of humane animal care approved by the Medical Ethics Committee of Oita University (No. R024001). BALB/c nu/nu female nude mice were purchased from Kyudo (Tosu, Japan). Injection of 1 × 10^6^ SKES-1 cells into the gluteal region of BALB/c nu/nu mice was performed Tumors were measured using calipers two or three times per week, as previously described^29^. Evaluation of mouse tumor volume after cells transfected with negative control-miR (20 nM), miR-152 mimic (20 nM), and CDK5R1-siRNA (20 nM) and untreated cells were injected subcutaneously into the left flank of each mouse (n = 5, 1 × 10^6^ cells/mouse). All the animals developed tumor within two weeks. Tumors were measured using calipers two or three times per week and the tumor volumes were calculated using the formula *V* = *ab*^2^/*2*, where *a* and *b* are the tumor length and width, respectively. Tumors were grown until they reached the volume of 30–50 mm^3^, following which the mice were randomly categorized into different groups and treated accordingly. The maximum tumor diameter permitted under relevant animal protocols is 25 mm, and this limit was not exceeded in any of the experiments. After quarantine, all mice were kept in a pathogen-free environment on a standard 12 h day/12 h night cycle and were fed a standard sterilized pellet diet and water ad libitum. After 5 weeks, the mice were then sacrificed by cervical dislocation and xenografted tumors were resected and subjected to immunohistochemistry.

### Statistical analysis

Two-tailed Student’s *t* test was performed for continuous variables. The differences among more than 3 groups were analyzed using ANOVA and Scheffe test. Results are expressed as mean ± standard deviation. Differences were considered significant when the p values were less than 0.05. All statistical analyses were performed using IBM SPSS Statistics version 24 (IBM Japan, Tokyo, Japan), as previously described^29^.Please note we have moved the sentence “All authors are responsible for the submission of this article and accept the conditions of submission” to the end of the Author Contributions, as per house style.I understand. Thank you for making the correction.

### Supplementary Information

Supplementary Information.

Supplementary Figures.

## Data Availability

All data analyzed during this study are included in this manuscript. The data generated in this study are publicly available in Gene Expression Omnibus (GEO) at GSE70827 and its URL is below: http://www.ncbi.nlm.nih.gov/geo/query/acc.cgi?acc=GSE70827.

## Abbreviations

7-AAD: 7-Amino-actinomycin D
ANOVA: Analysis of variance
BrdU: 5-Bromo-2-deoxyuridine
CCNA: Cyclin A
CCNB: Cyclin B
CCND: Cyclin D
CCNE: Cyclin E
CDC: Cell division cycle
CDK5R1: Cyclin-dependent kinase-5 activator 1
CDK: Cyclin dependent kinase
ETS: E26 transformation-specific
FITC: Fluorescein isothiocyanate
GAPDH: Glyceraldehyde- 3-phosphate dehydrogenase
IP: Immunoprecipitation
PARP: Poly ADP-ribose polymerase
PBS: Phosphate-buffered saline
