# Supplementary figures and images for: Tumor-suppressive microRNA-152 inhibits the proliferation of Ewing’s sarcoma cells by targeting CDK5R1
Source: Sci Rep. 2023 Oct 29;13:18546. doi: 10.1038/s41598-023-45833-6 (PMC10613623; doi:10.1038/s41598-023-45833-6)

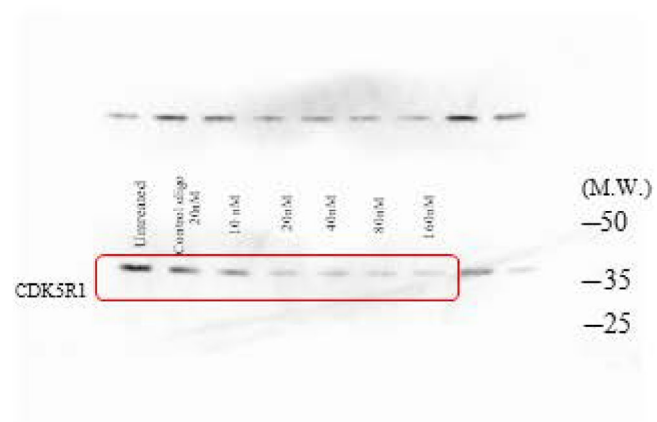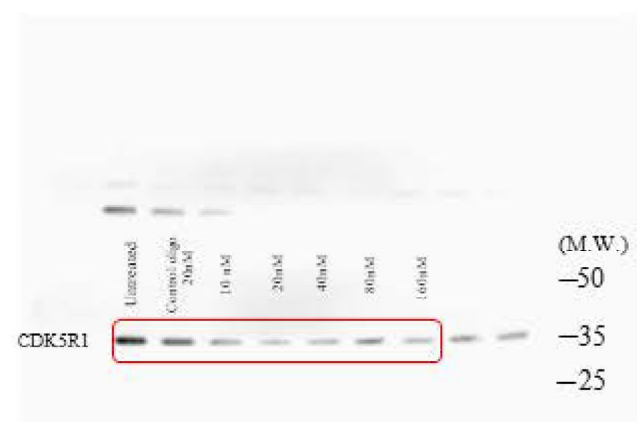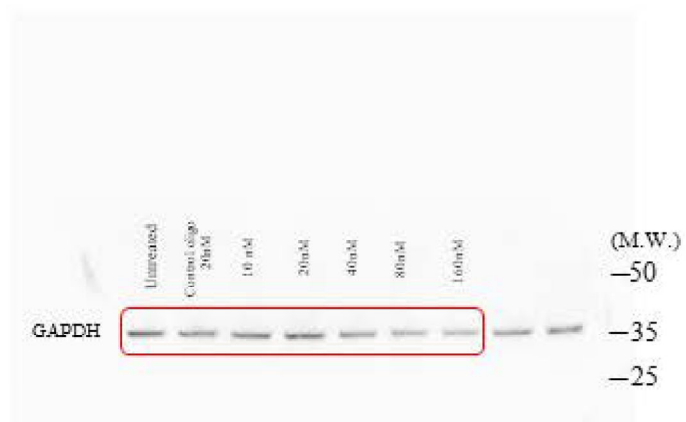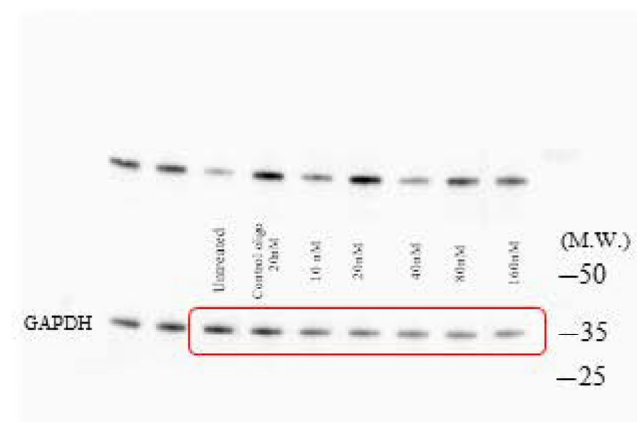

Fig. 1

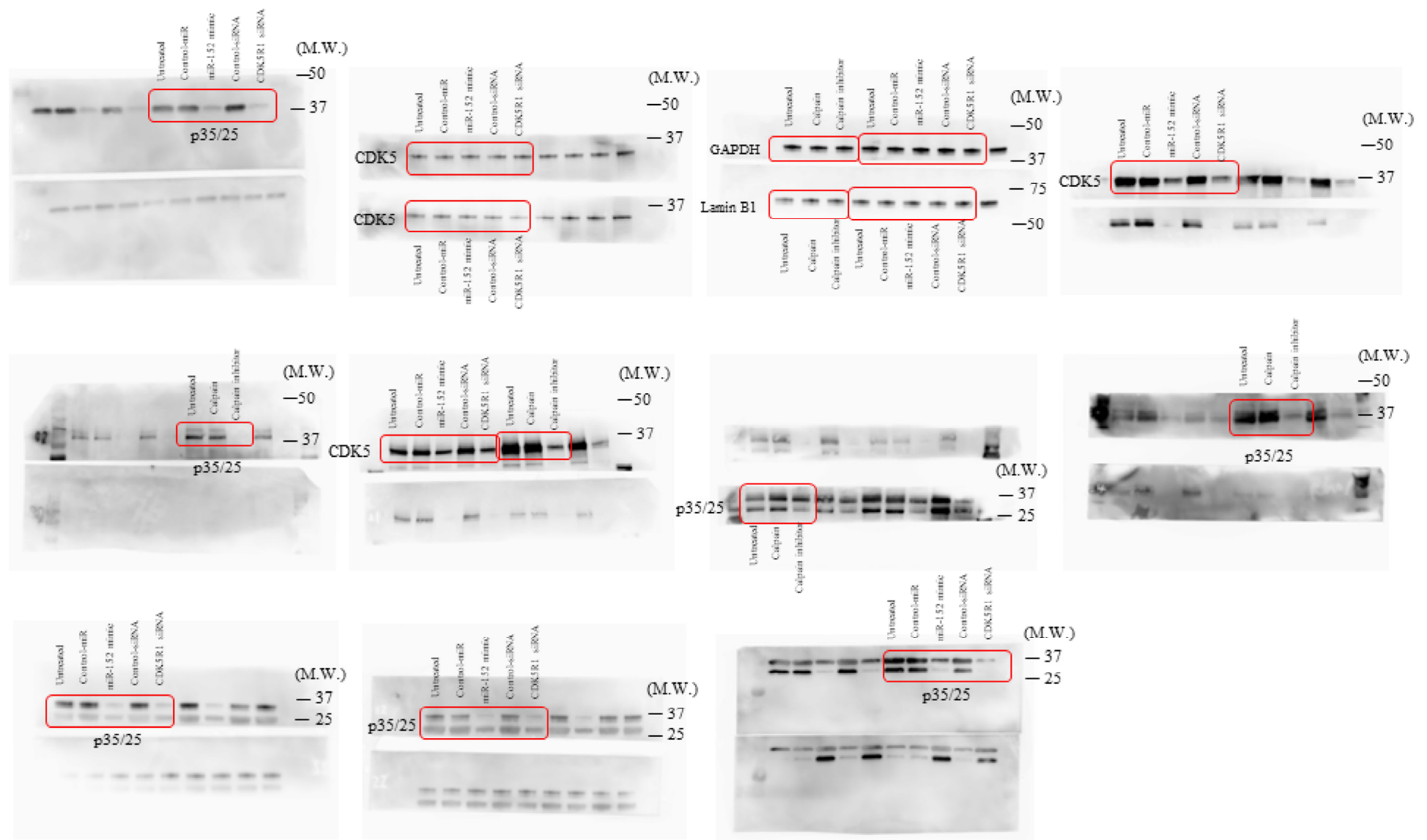

Fig.2.

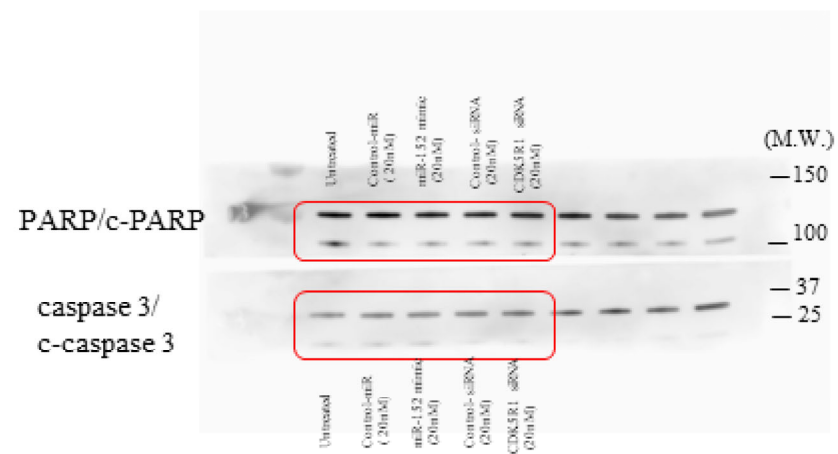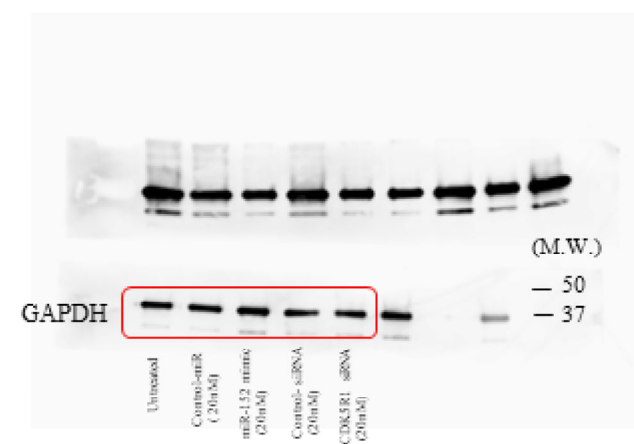

Fig.3.

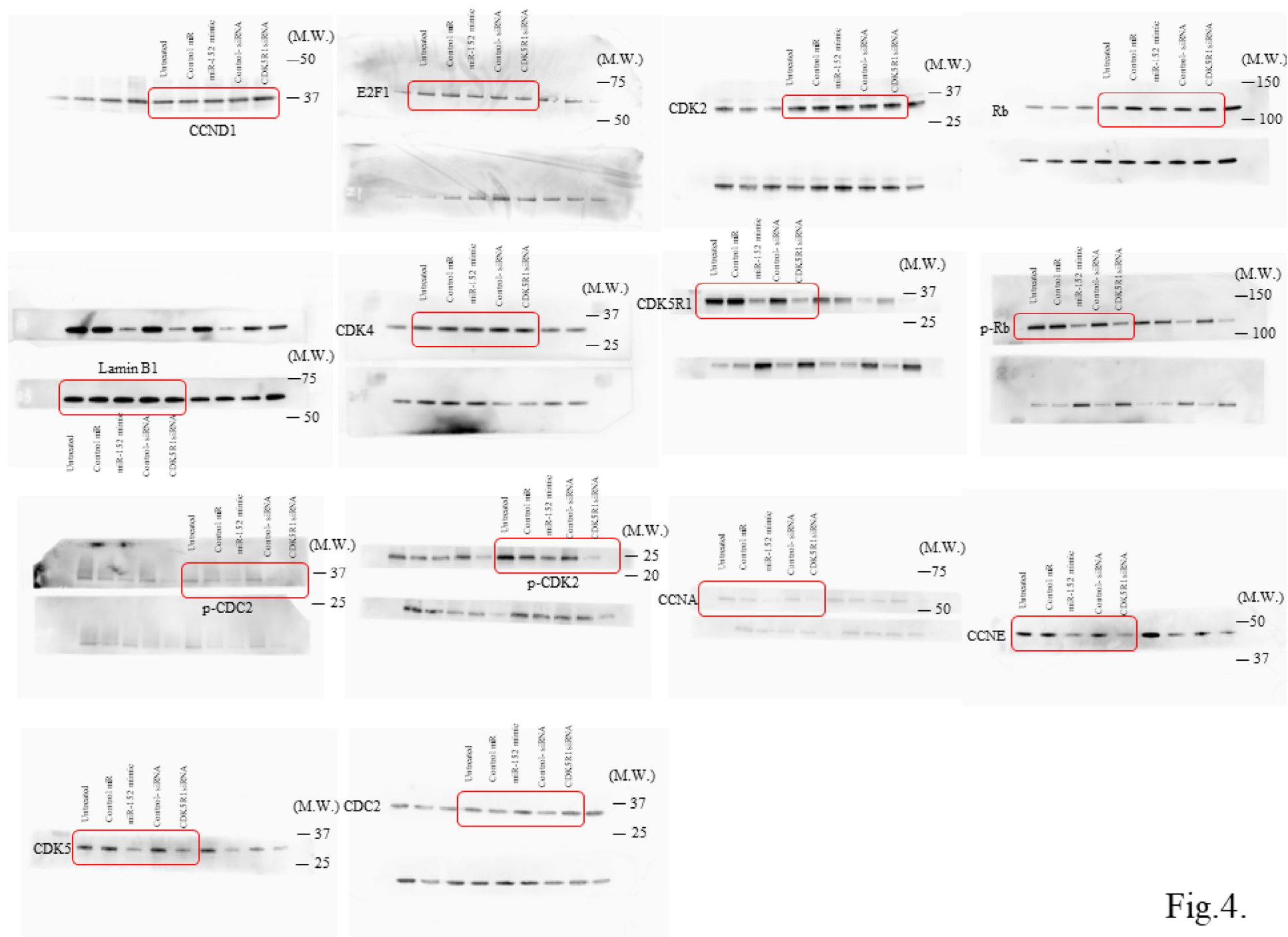

Fig.4.

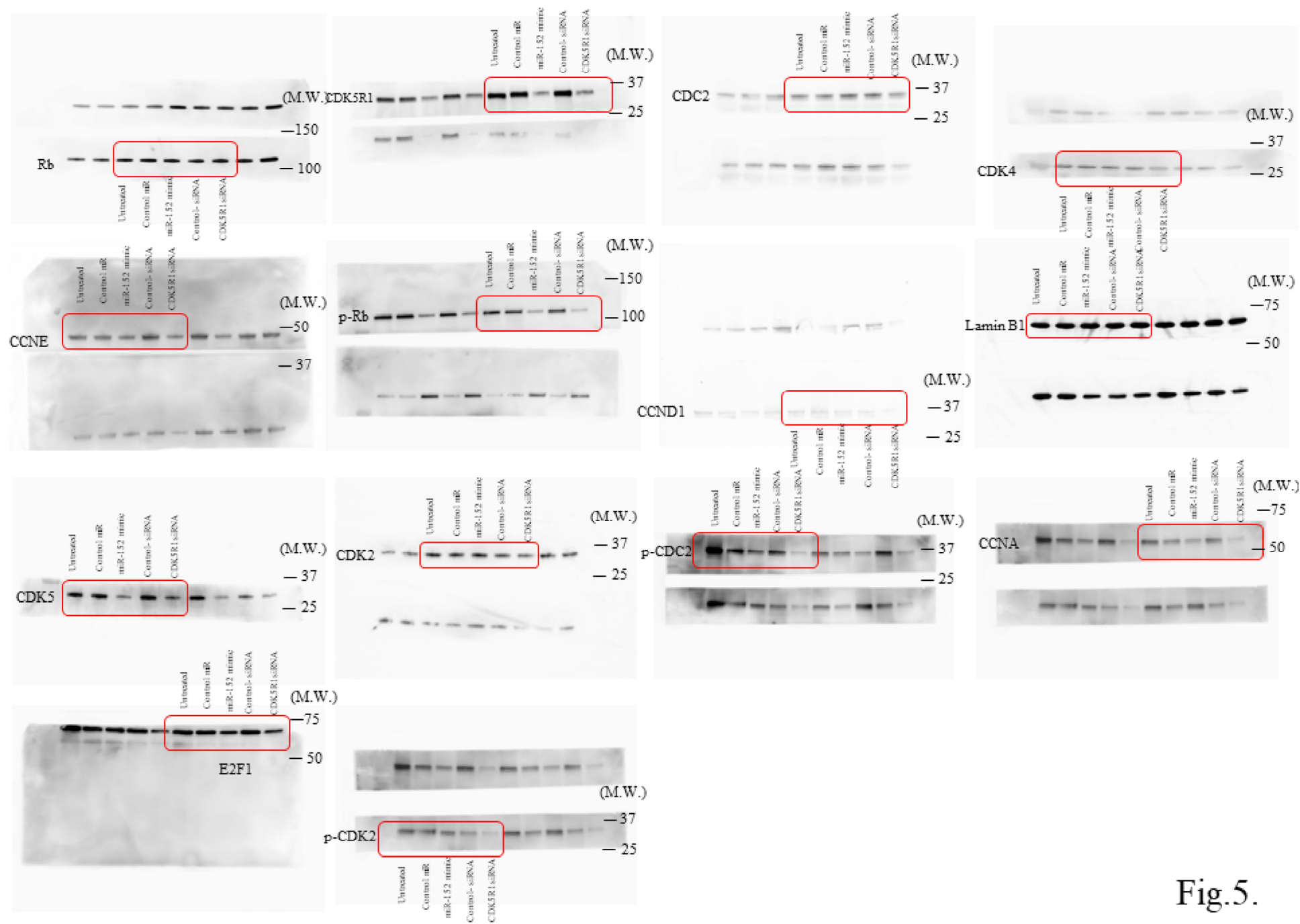

Fig.5.

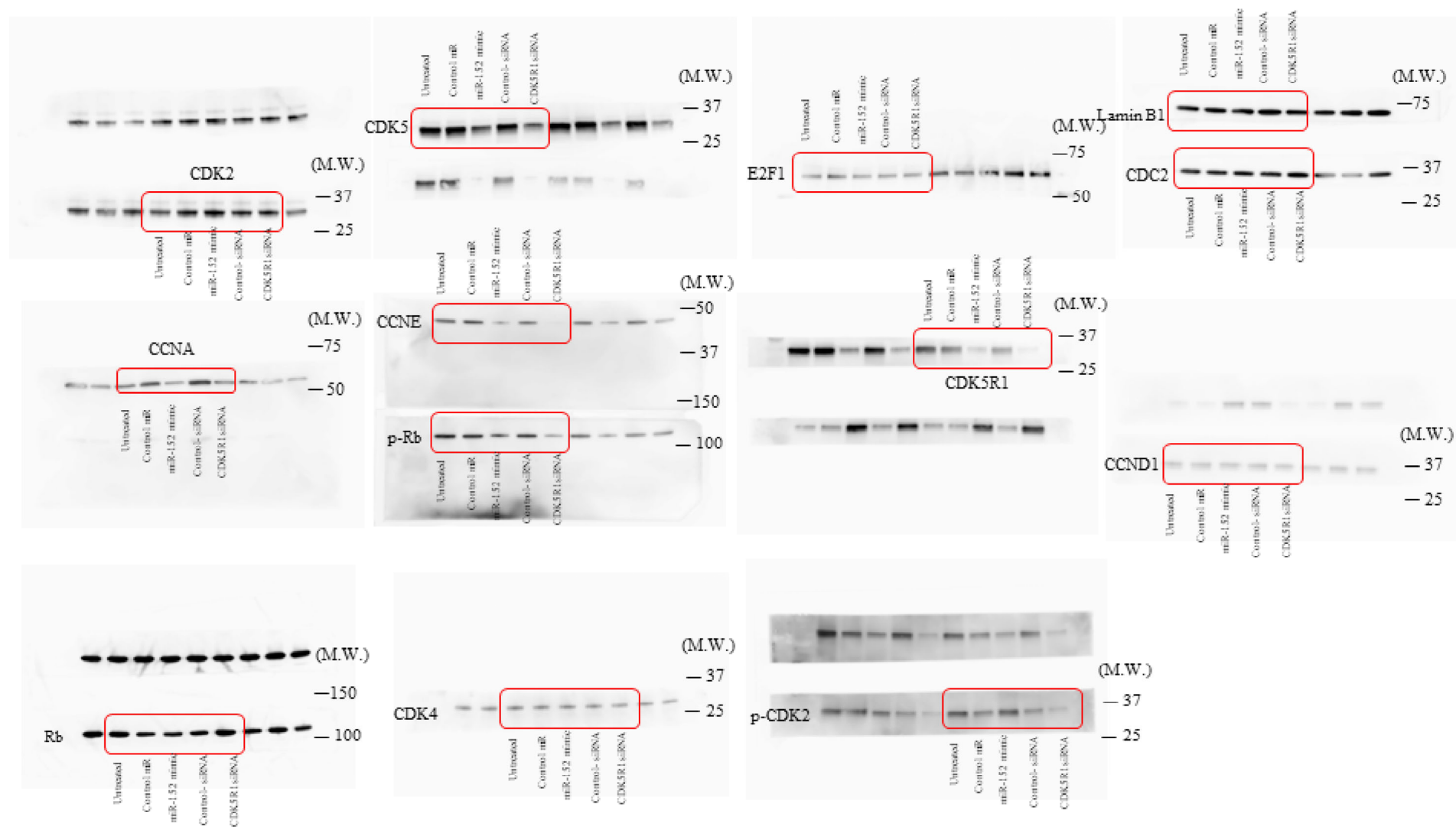

Fig.6.

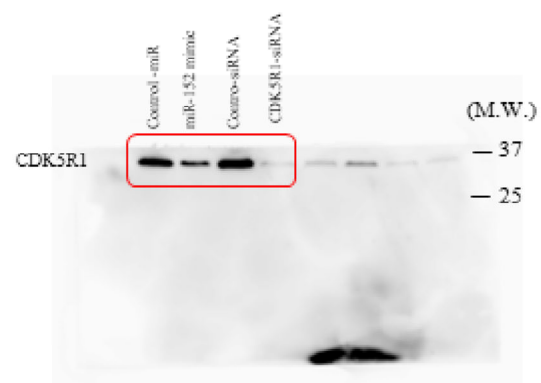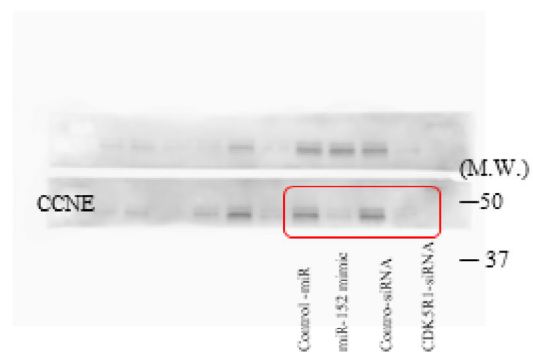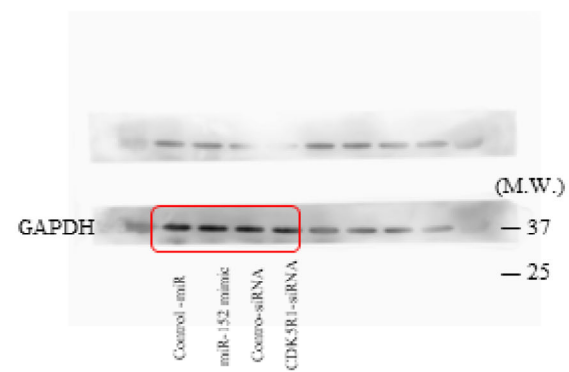

Fig.7.

Supplement: Supplementary file 1 — Supplementary Information. [file 41598_2023_45833_MOESM1_ESM.pdf]
