# Supplementary material for: Tumor-suppressive microRNA-152 inhibits the proliferation of Ewing’s sarcoma cells by targeting CDK5R1
Source: Sci Rep. 2023 Oct 29;13:18546. doi: 10.1038/s41598-023-45833-6 (PMC10613623; doi:10.1038/s41598-023-45833-6)
Supplement: Supplementary file 2 — Supplementary Figures. [file 41598_2023_45833_MOESM2_ESM.pdf]

Figure S1

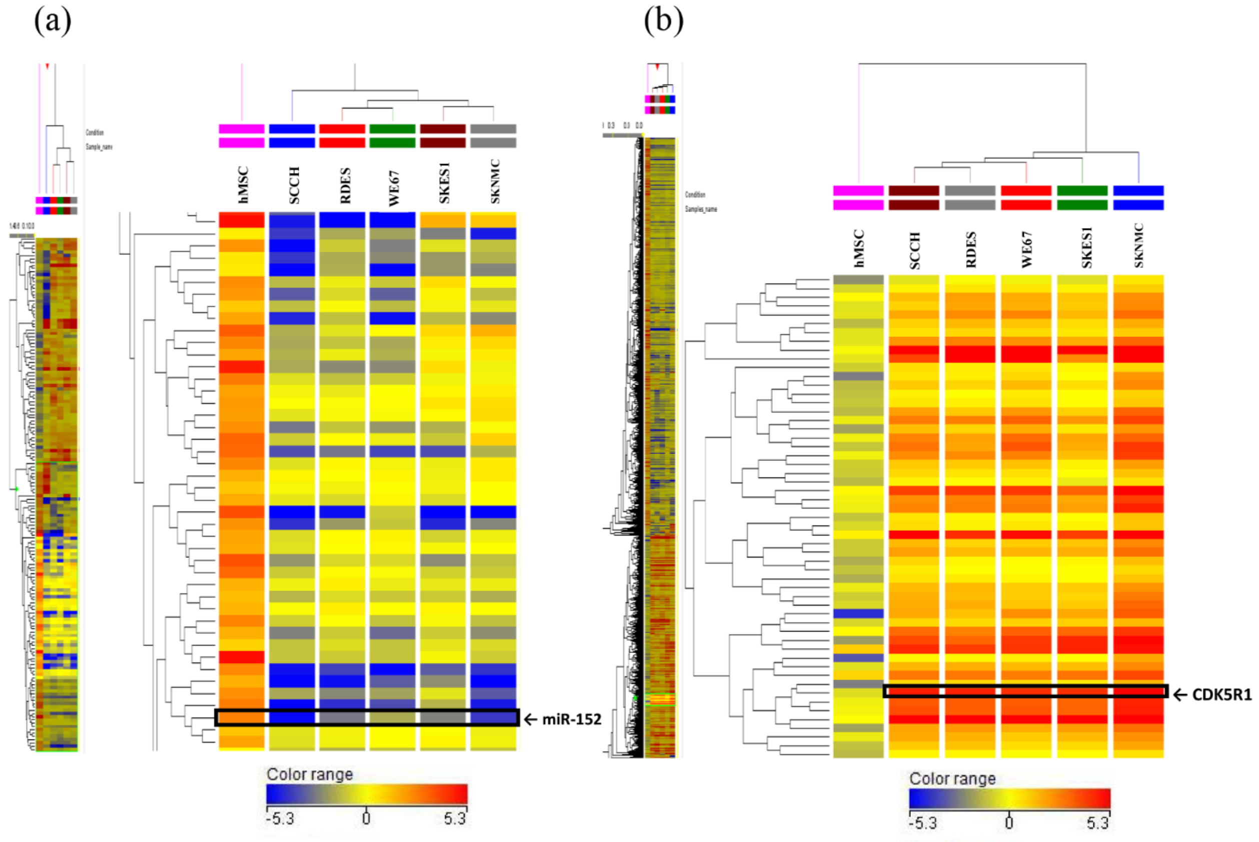

Supplementary Fig. S1: Down regulation of miR-152 and up regulation of CDK5R1 expression in ES cell lines. (a) The genome-wide miRNA expression profiling using five ES cell lines was carried out to identify miRNAs specifically expressed in ES cells. The array analysis showed that the expressions of 1054 miRNAs in ES cells were changed (fold-change >2.0) in comparison with hMSCs. Among 1054 miRNAs, 228 were significantly up-regulated, whereas 705 were significantly down-regulated in all tested ES cells compared to hMSCs. The remaining 121 miRNAs were up or down regulated among five ES cell lines. **(b)** The cDNA array analysis demonstrated that the expressions of 3043 mRNAs were significantly changed between five ES cell lines and hMSCs. We found that 1062 genes were significantly up-regulated, whereas 1884 genes were significantly down regulated and the remaining 97 genes were up or down regulated in five ES cell lines compared to hMSCs.

Figure S2

(a)

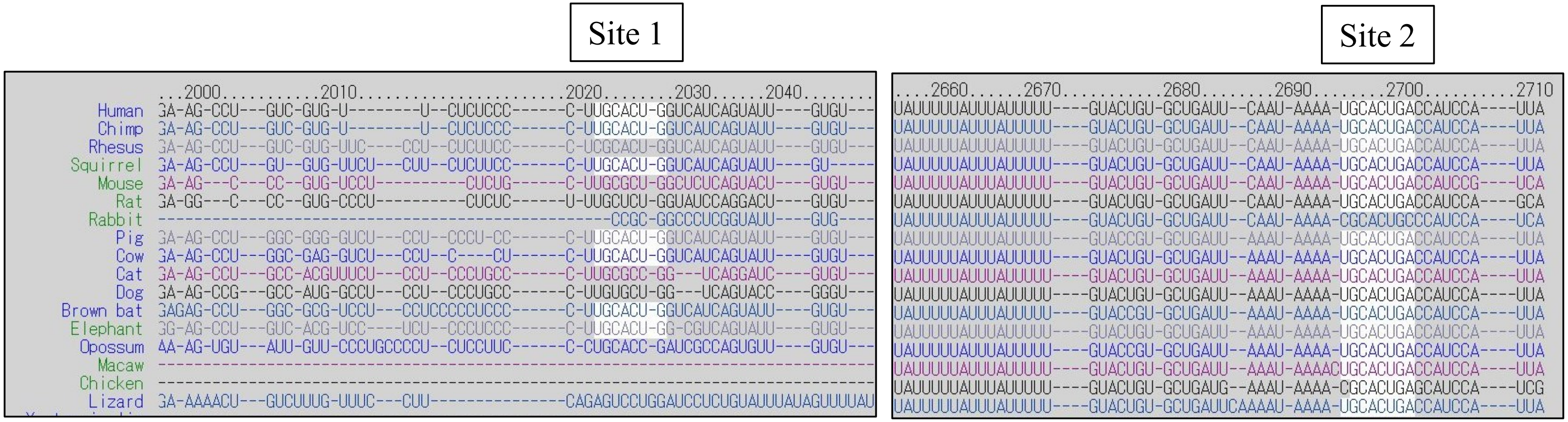

(b)

	Predicted consequential pairing of target region (top) and miRNA (bottom)	Site type	Context++ score	Context++ score percentile	Weighted context++ score	Conserved branch length	P_{CT}	Predicted relative K_D
Position 2022-2028 of CDK5R1 3' UTR<br>hsa-miR-152-3p	5' ...CGUGUUCUCUCCCCUUGCACUGG...<br>3' GGUUCAAGACAGU---ACGUGACU	7mer-m8	-0.19	84	-0.19	3.605	0.42	-4.551
Position 2695-2702 of CDK5R1 3' UTR<br>hsa-miR-152-3p	5' ...GCUGAUUCAAUAAAAUGCACUGA...<br>3' GGUUCAAGACAGU--ACGUGACU	8mer	-0.55	99	-0.54	5.307	0.88	-5.891

Supplementary Figure S2: Prediction and Binding Affinity Assessment of has-miR-152-3p and CDK5R1 Binding Sites using Target Scan Human 8.0. (a) Predicted binding sites of CDK5R1 and miR-152-3p according to Target Scan 8.0 are shown. Investigation of CDK5R1's 3'-UTR indicated two potential binding sites for has-miR-152-3p (MIMAT0000438). **(b)** The binding affinity between CDK5R1 and has-miR-152-3p is illustrated by the Context++ score. The Context++ score represents affinity within the range of -1.0 to 1.0, with lower values indicating higher affinity. The score for Sites 1 and 2 is -0.19 and -0.55, respectively, suggesting strong binding affinity, especially at site 2.

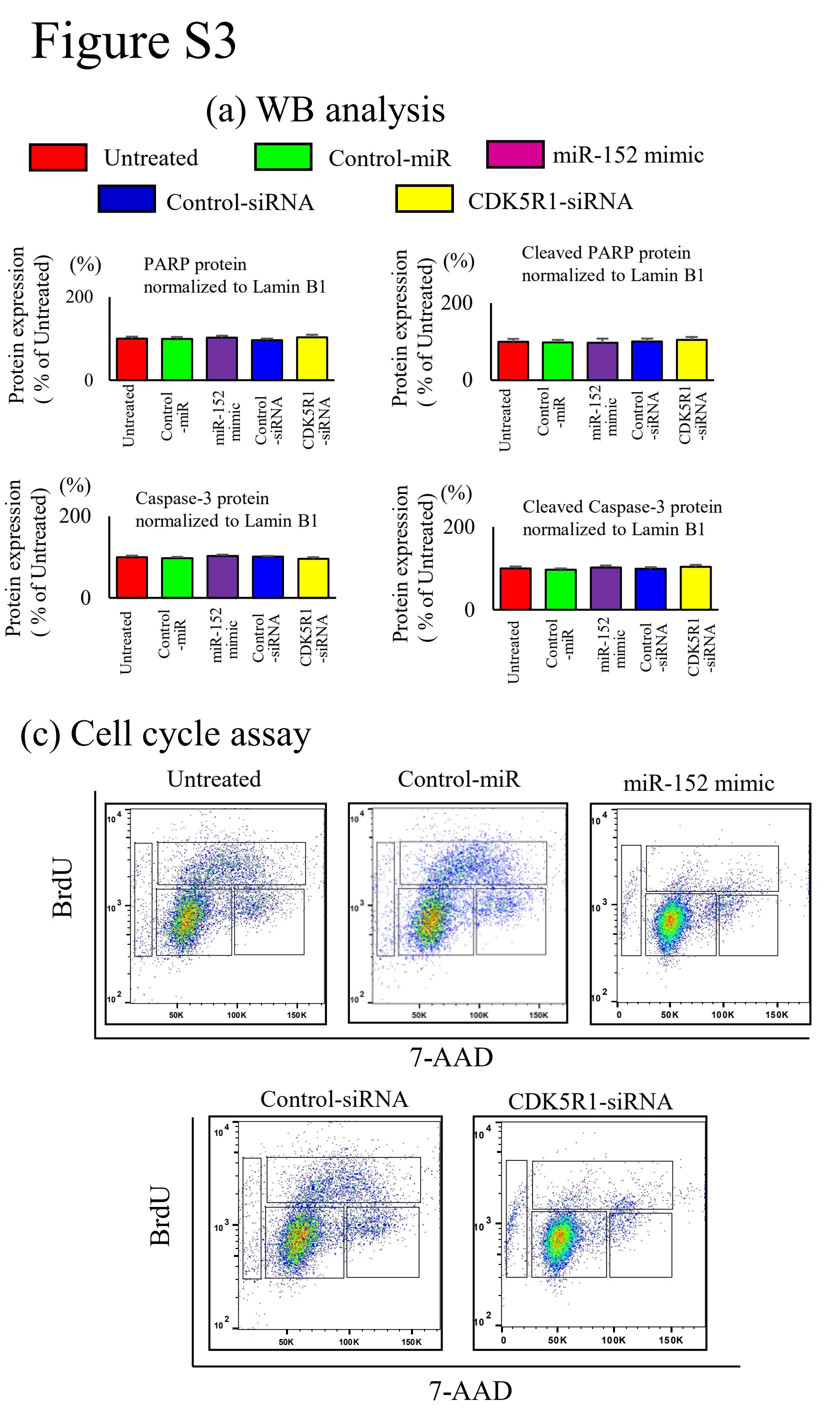

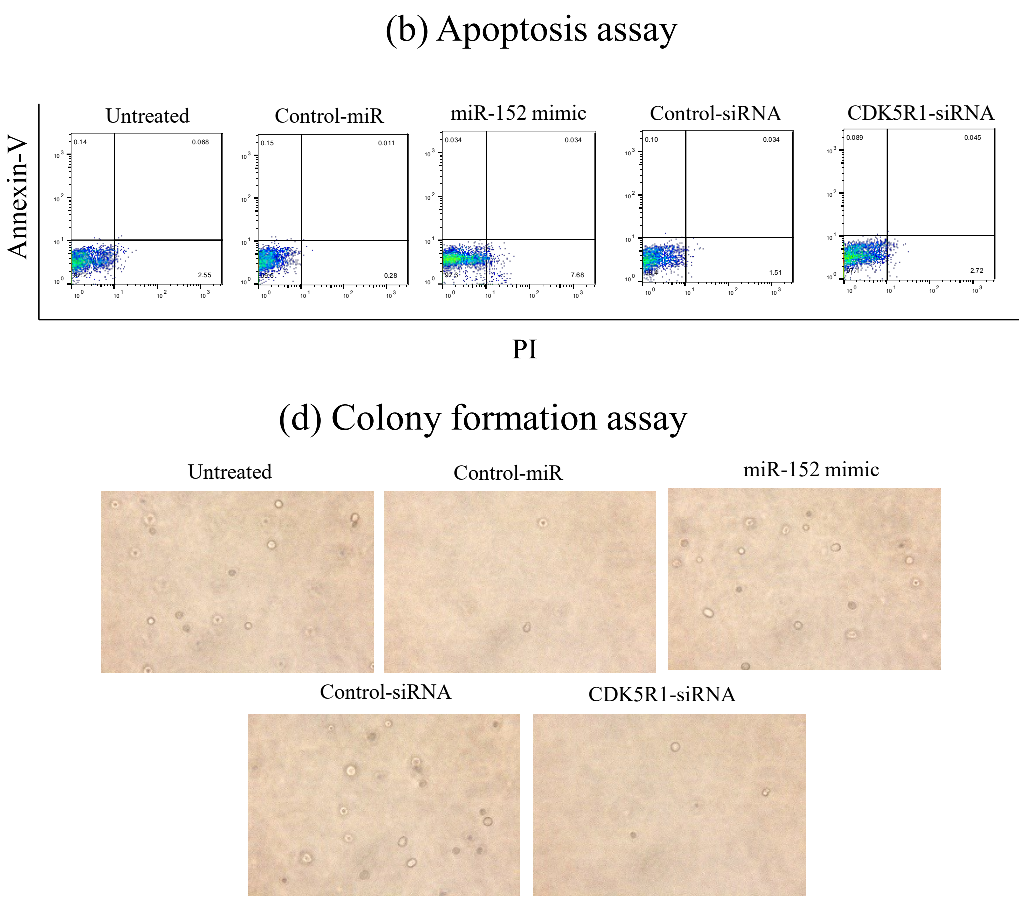

Supplementary Figure S3: Impact of miR-152 transfection and CDK5R1 knockdown on Cell Proliferation. (a) Apoptosis assessment was conducted based on the expression of cleaved PARP and cleaved caspase-3. **(b)** Apoptosis evaluation was performed using Annexin-V on the vertical axis and PI staining on the horizontal axis. **(c)** Cell cycle analysis was conducted by dual staining with BrdU on the vertical axis and 7-AAD on the horizontal axis. **(d)** Microphotographs, taken using phase contrast microscopy with a ×20 magnification objective, show representative images of the different conditions of soft agar colony formation.

Figure S4

(a) WB analysis

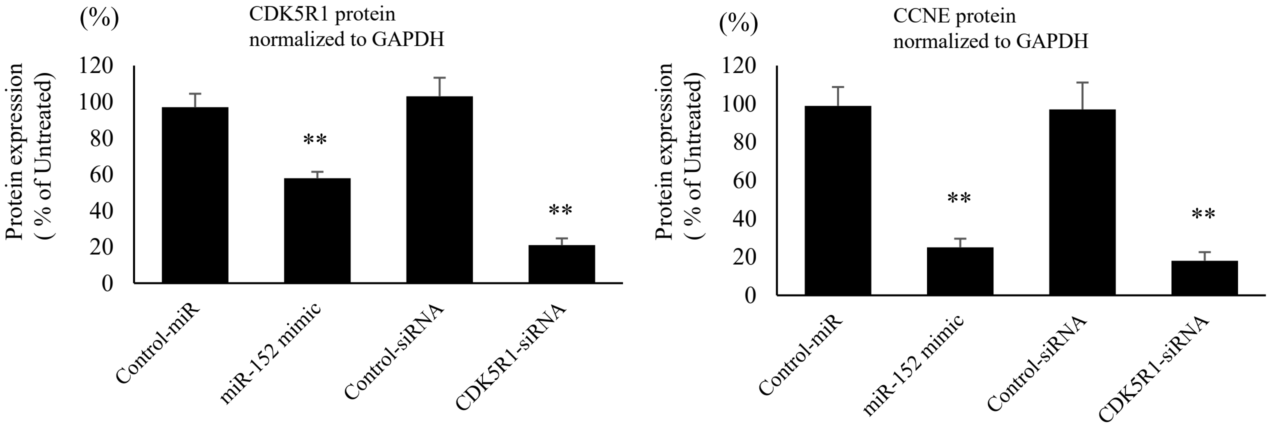

(b) Positive cells

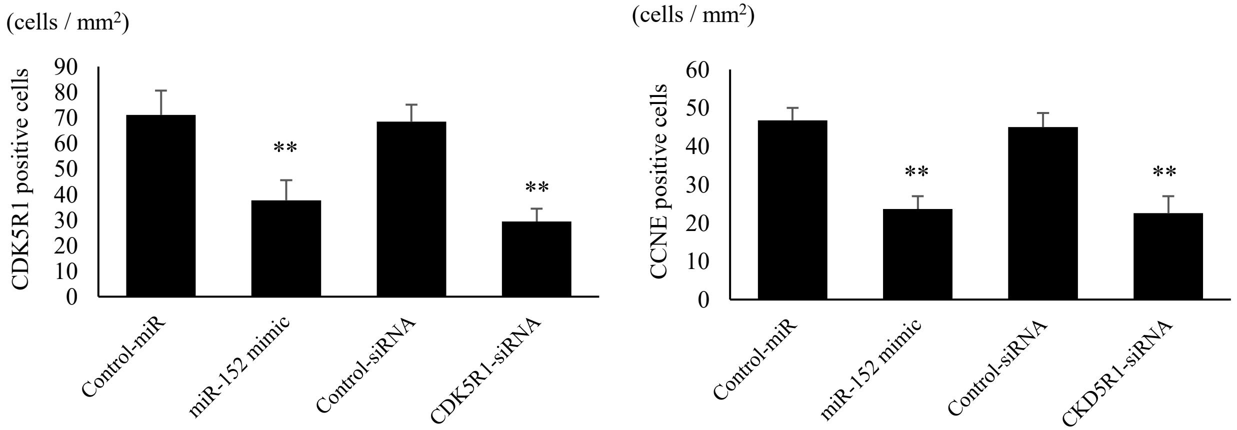

Supplementary Figure S4: Expression of CDK5R1 and CCNE in Mouse Tumor Tissues. (a) Protein extraction from mouse tumor tissues was performed, and the expression of CDK5R1 and CCNE was investigated through Western blot analysis. **(b)** Expression of CDK5R1 and CCNE in mouse tumor tissues was evaluated using immunohistochemical staining.
